# COVID-19 vaccine acceptance and hesitancy in low- and middle-income countries

**DOI:** 10.1038/s41591-021-01454-y

**Published:** 2021-07-16

**Authors:** Julio S. Solís Arce, Shana S. Warren, Niccolò F. Meriggi, Alexandra Scacco, Nina McMurry, Maarten Voors, Georgiy Syunyaev, Amyn Abdul Malik, Samya Aboutajdine, Opeyemi Adeojo, Deborah Anigo, Alex Armand, Saher Asad, Martin Atyera, Britta Augsburg, Manisha Awasthi, Gloria Eden Ayesiga, Antonella Bancalari, Martina Björkman Nyqvist, Ekaterina Borisova, Constantin Manuel Bosancianu, Magarita Rosa Cabra García, Ali Cheema, Elliott Collins, Filippo Cuccaro, Ahsan Zia Farooqi, Tatheer Fatima, Mattia Fracchia, Mery Len Galindo Soria, Andrea Guariso, Ali Hasanain, Sofía Jaramillo, Sellu Kallon, Anthony Kamwesigye, Arjun Kharel, Sarah Kreps, Madison Levine, Rebecca Littman, Mohammad Malik, Gisele Manirabaruta, Jean Léodomir Habarimana Mfura, Fatoma Momoh, Alberto Mucauque, Imamo Mussa, Jean Aime Nsabimana, Isaac Obara, María Juliana Otálora, Béchir Wendemi Ouédraogo, Touba Bakary Pare, Melina R. Platas, Laura Polanco, Javaeria Ashraf Qureshi, Mariam Raheem, Vasudha Ramakrishna, Ismail Rendrá, Taimur Shah, Sarene Eyla Shaked, Jacob N. Shapiro, Jakob Svensson, Ahsan Tariq, Achille Mignondo Tchibozo, Hamid Ali Tiwana, Bhartendu Trivedi, Corey Vernot, Pedro C. Vicente, Laurin B. Weissinger, Basit Zafar, Baobao Zhang, Dean Karlan, Michael Callen, Matthieu Teachout, Macartan Humphreys, Ahmed Mushfiq Mobarak, Saad B. Omer

**Affiliations:** 1 WZB Berlin Social Science Center, Berlin, Germany; 2 Innovations for Poverty Action (IPA), New York, NY USA; 3 International Growth Centre (IGC), Freetown, Sierra Leone; 4 Wageningen University & Research, Wageningen, the Netherlands; 5 International Center for the Study of Institutions and Development (ICSID), HSE University, Moscow, Russia; 6 Columbia University, New York, NY USA; 7 Yale Institute for Global Health, New Haven, CT USA; 8 Busara Center for Behavioral Economics, Lagos, Nigeria; 9 Department of Sociology, University of Lagos, Lagos, Nigeria; 10 Busara Nigeria, Lagos, Nigeria; 11 Agricultural and Rural Development Secretariat, Federal Capital Territory Administration, Abuja, Nigeria; 12 Nova School of Business and Economics, Lisbon, Portugal; 13 The Institute for Fiscal Studies, London, UK; 14 Lahore University of Management Sciences, Lahore, Pakistan; 15 Innovations for Poverty Action (IPA) Uganda, Kampala, Uganda; 16 Morsel Research & Development, Lucknow, India; 17 University of St Andrews, St Andrews, UK; 18 Redes Peru, Lima, Peru; 19 Stockholm School of Economics and Misum, Stockholm, Sweden; 20 Ghent University, Department of Economics, Ghent, Belgium; 21 Innovations for Poverty Action (IPA) Colombia, Bogotá, Colombia; 22 Institute of Development and Economic Alternatives, Lahore, Pakistan; 23 Innovations for Poverty Action (IPA) Sierra Leone, Freetown, Sierra Leone; 24 NOVAFRICA, Lisbon, Portugal; 25 Trinity College Dublin, Dublin, Ireland; 26 Institute of Public Administration and Management, University of Sierra Leone, Freetown, Sierra Leone; 27 Centre for the Study of Labour and Mobility (CESLAM), Kathmandu, Nepal; 28 Cornell University, Ithaca, NY USA; 29 University of Illinois Chicago, Chicago, IL USA; 30 Innovations for Poverty Action (IPA) Rwanda, Kigali, Rwanda; 31 Associação NOVAFRICA para o Desenvolvimento Empresarial e Económico de Moçambique, Maputo, Mozambique; 32 Innovations for Poverty Action (IPA) Burkina Faso, Ouagadougou, Burkina Faso; 33 NYU Abu Dhabi, Abu Dhabi, United Arab Emirates; 34 Centre for Economic Research in Pakistan (CERP), Lahore, Pakistan; 35 Yale Research Initiative on Innovation and Scale (Y-RISE), New Haven, CT USA; 36 Princeton University, Princeton, NJ USA; 37 Institute for International Economic Studies (IIES), Stockholm University, Stockholm, Sweden; 38 Tufts University, Medford, MA USA; 39 University of Michigan, Ann Arbor, MI USA; 40 Kellogg School of Management at Northwestern University, Evanston, IL USA; 41 London School of Economics and Political Science, London, UK; 42 Yale University, New Haven, CT USA

**Keywords:** Epidemiology, Interdisciplinary studies

## Abstract

Widespread acceptance of COVID-19 vaccines is crucial for achieving sufficient immunization coverage to end the global pandemic, yet few studies have investigated COVID-19 vaccination attitudes in lower-income countries, where large-scale vaccination is just beginning. We analyze COVID-19 vaccine acceptance across 15 survey samples covering 10 low- and middle-income countries (LMICs) in Asia, Africa and South America, Russia (an upper-middle-income country) and the United States, including a total of 44,260 individuals. We find considerably higher willingness to take a COVID-19 vaccine in our LMIC samples (mean 80.3%; median 78%; range 30.1 percentage points) compared with the United States (mean 64.6%) and Russia (mean 30.4%). Vaccine acceptance in LMICs is primarily explained by an interest in personal protection against COVID-19, while concern about side effects is the most common reason for hesitancy. Health workers are the most trusted sources of guidance about COVID-19 vaccines. Evidence from this sample of LMICs suggests that prioritizing vaccine distribution to the Global South should yield high returns in advancing global immunization coverage. Vaccination campaigns should focus on translating the high levels of stated acceptance into actual uptake. Messages highlighting vaccine efficacy and safety, delivered by healthcare workers, could be effective for addressing any remaining hesitancy in the analyzed LMICs.

## Abstract (web summary)

Survey data collected across ten low-income and middle-income countries (LMICs) in Asia, Africa and South America compared with surveys from Russia and the United States reveal heterogeneity in vaccine confidence in LMICs, with healthcare providers being trusted sources of information, as well as greater levels of vaccine acceptance in these countries than in Russia and the United States.

## Main

A safe and effective vaccine is a critical tool to control the COVID-19 pandemic. As of 25 June 2021, 23 vaccines had advanced to Stage 3 clinical trials^1^ and more than a dozen had been approved in multiple countries^2^. The BNT162b vaccine from Pfizer–BioNTech, for example, has been approved in about 90 countries, while the ChAdOx1 nCoV-19 vaccine from Oxford–AstraZeneca has the most country authorizations at 115^2^. At present, however, global vaccine distribution remains highly unequal, with much of the current supply directed toward high-income countries^3^.

Although effective and equitable distribution of COVID-19 vaccines is a key policy priority, ensuring acceptance is just as important. Trust in vaccines as well as the institutions that administer them are key determinants of the success of any vaccination campaign^4^. Several studies have investigated willingness to take a potential COVID-19 vaccine in high-income countries^5–10^, and some studies have included middle-income countries^3,11^. Less is known, however, about vaccine acceptance in low-income countries where large-scale vaccination has yet to begin. Understanding the drivers of COVID-19 vaccine acceptance is of global concern, because a lag in vaccination in any country may result in the emergence and spread of new variants that can overcome immunity conferred by vaccines and prior disease^12,13^.

Our study complements the emerging global picture of COVID-19 vaccine acceptance by focusing primarily on lower-income countries. We construct a sample of low- and middle-income countries (LMICs) with wide geographic coverage across Africa, Asia and Latin America. We move beyond documenting vaccine acceptance rates to collect and analyze data on the reasons for acceptance and hesitancy, which is critical for informing the design of effective vaccine distribution and messaging. A summary of the main findings, limitations and implications of the study is shown in Table 1.

**Table 1:** Policy summary

Background	We analyze COVID-19 vaccine acceptance and hesitancy and their drivers across 15 survey samples covering 10 LMICs in Asia, Africa and South America, as well as Russia and the United States, comprising a total of 44,260 individuals.
Main findings and limitations	Willingness to take a COVID-19 vaccine is considerably higher in the LMICs in our sample than in the United States and Russia. The personal protective benefit of vaccination is the most frequently cited reason for vaccine acceptance. Concern about side effects is the most commonly cited reason for vaccine hesitancy. Health workers are considered the most trusted sources of guidance about COVID-19 vaccine choices. One limitation of our study is that our data are not representative of all LMICs, and some individual samples are not nationally representative. However, our main findings—of high COVID-19 vaccine acceptance in our LMIC samples relative to the United States and Russia—are consistent across samples and specifications.
Policy implications	Although global vaccine distribution has skewed heavily toward higher-income countries so far, the high levels of vaccine acceptance we identify suggest that prioritizing distribution to LMICs may be an efficient way to achieve immunity on a global scale and prevent novel variants from emerging. Vaccination campaigns should focus on converting positive intentions into uptake, which may require investment in local supply chains and delivery. Engaging health workers to deliver vaccine information, leveraging pro-vaccine norms, and messaging focused on vaccine effectiveness and safety might be effective in addressing remaining hesitancy.

Acceptance of childhood vaccination for common diseases—such as measles (MCV), Bacille Calmette–Guérin (BCG) and diphtheria, tetanus and pertussis (DTP)—is generally high in LMICs, providing grounds for optimism about the prospects for COVID-19 vaccine uptake. Table 2 summarizes general vaccine acceptance^14^ and coverage rates of childhood vaccines in 2018^15^, prior to the current pandemic, for the countries included in our study. Agreement on the importance of childhood vaccinations is markedly higher in the LMICs we study compared to Russia and the United States. However, existing studies on COVID-19 vaccine acceptance document substantial variation, both across and within countries, including in settings with high acceptance of other vaccinations^3,4,11^.

**Table 2:** Vaccination beliefs and coverage for the countries studied

	Effective	Safe	Important for children to have	Tuberculosis (BCG)	Diphtheria, tetanus and pertussis (DTP1)	Measles (MCV1)	Percent of parents with any child that was ever vaccinated
Burkina Faso	87	72	95	98	95	88	97
Colombia	83	84	99	89	92	95	95
India	96	97	98	92	94	95	92
Mozambique	87	93	98	94	93	87	95
Nepal	89	93	99	96	96	92	95
Nigeria	82	92	96	67	65	54	95
Pakistan	91	92	95	88	86	75	94
Rwanda	99	97	99	98	99	96	100
Sierra Leone	95	95	99	86	95	93	97
Uganda	82	87	98	88	99	87	98
Russia	67	48	80	96	97	98	96
United States	85	73	87	–	97	90	95

The table presents an overview of vaccination beliefs and incidence across countries in our sample. Columns 2–4 and 8 use data from the Wellcome Global Monitor 2018^14^. Column 8 shows the percentage of respondents who are parents and report having had any of their children ever vaccinated. Columns 2–4 show the percentage of all respondents that either strongly agree or somewhat agree with the statement above each column. All percentages are obtained using national weights. Columns 5–7 use data from the World Health Organization on vaccine incidence^15^. Columns 5–7 report the percentage of infants per country receiving the vaccine indicated in each column.

The existing literature cites concern about COVID-19 vaccine safety, including the rapid pace of vaccine development, as a primary reason for hesitancy in higher-income settings^3,5^. Other reasons may feature more prominently in LMICs. For example, reported COVID-19 cases and deaths have been consistently lower in most LMICs relative to higher-income countries^16–18^. If individuals feel the risk of disease is less severe, they may be less willing to accept any perceived risks of vaccination^19^. Previous studies of healthcare utilization in LMICs have also highlighted factors such as negative perceptions of healthcare quality^20^, negative historical experiences involving foreign actors^21,22^, weak support from traditional leaders^23^ and mistrust in government^24^ as barriers to uptake, which could apply to COVID-19 vaccination as well.

## Results

To promote vaccination against COVID-19, we need to know whether people are willing to take COVID-19 vaccines, the reasons why they are willing or unwilling to do so, and the most trusted sources of information in their decision-making. Our study investigates these questions using a common set of survey items deployed across 13 studies in Africa, South Asia and Latin America (Table 3): seven surveys in low-income countries (Burkina Faso, Mozambique, Rwanda, Sierra Leone and Uganda), five surveys in lower-middle-income countries (India, Nepal, Nigeria and Pakistan) and one in an upper-middle-income country (Colombia). We compare these findings to those from two countries at the forefront of vaccine research and development, Russia (upper-middle income) and the United States (high income).

**Table 3:** Summary of study sampling protocols

Study	Date	Geographic scope	Sampling methodology	Survey modality	Weights
Burkina Faso	15 October to 4 December 2020	National	RDD	Phone	Yes
Colombia	15–25 August 2020	National	RDD	Phone	Yes
India	17 June 2020 to 18 January 2021	Subnational, slums in two cities	Representative sample of slum dwellers living in the vicinity of a community toilet and located in Uttar Pradesh	Phone	Yes
Mozambique	30 October to 30 November 2020	Subnational, two cities	(1) Random sample in urban and peri-urban markets stratified by gender and type of establishment in Maputo; (2) random sample representative of communities in the Cabo Delgado, stratified on urban, semi-urban and rural areas	Phone	No
Nepal	1–11 December 2020	Subnational, two districts	Random sample of poor households from randomly selected villages in Kanchanpur	Phone	Yes
Nigeria	18 November to 18 December 2020	Subnational, one state	(1) Random sample of individuals in Kaduna; (2) sample of phone numbers from a phone list of Kaduna state residents	Phone	No
Pakistan 1	24 July to 9 September 2020	Subnational, two districts	Random sample of individuals in administrative police units in two districts of Punjab	Phone	Yes
Pakistan 2	2 September to 13 October 2020	Subnational, one province	RDD on a random sample of all numerically possible mobile phone numbers in the region of Punjab	Phone	No
Russia	6 November to 1 December 2020	Subnational, 61 regions	Sample recruited from the Russian online survey company OMI (Online Market Intelligence); sampling was targeted at having a minimum of 150 respondents per region, as well as representation of age, gender and education group	Online	Yes
Rwanda	22 October to 15 November 2020	National	RDD	Phone	Yes
Sierra Leone 1	2–19 October 2020	National	RDD	Phone	Yes
Sierra Leone 2	7 October 2020 to 20 January 2021	National	Random sample of households in 195 rural towns across all 14 districts of Sierra Leone	Phone	No
Uganda 1	21 September to 6 December 2020	Subnational, 13 districts	Sample of women in households from semi-rural and rural villages across 13 districts in Uganda, selected according to the likelihood of having children	Phone	No
Uganda 2	23 November to 12 December 2020	Subnational, one district	Random sample of households in Kampala	Phone	No
United States	4–5 December 2020	National	Nationwide sample of adult internet users recruited through the market research firm Lucid	Online	Yes

RDD, random digit dialing.

To select studies to include in our sample, we conducted an internal search within Innovations for Poverty Action (IPA), the International Growth Center (IGC) and the Berlin Social Science Center (WZB) for projects with plans to collect survey data in the second half of 2020. Study investigators agreed to include a set of common questions about COVID-19 vaccine attitudes. This strategy was guided by the need to collect information quickly and cost-effectively using a survey modality (phone) that was both safe, given pandemic conditions, and appropriate for contexts with limited internet coverage. The final set of samples included in our study therefore reflects populations that fall under the current research priorities at IGC, IPA and WZB and, in the case of IPA and IGC, donors that prioritize working in the Global South.

**Fig. 1:**
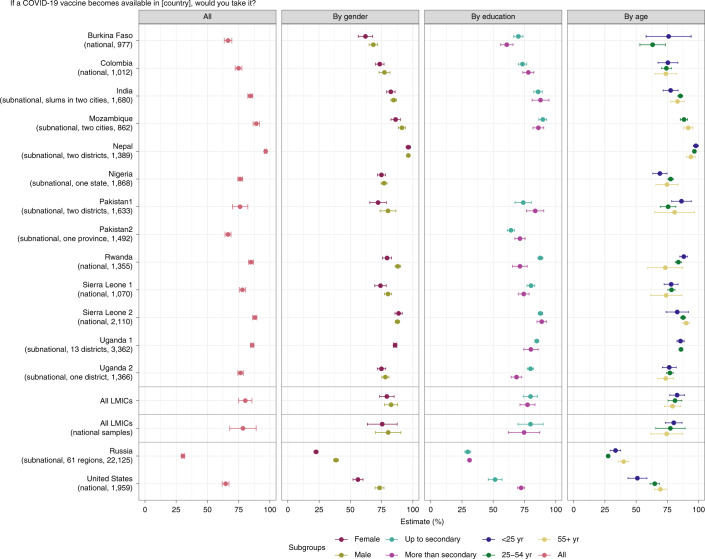
Acceptance rates, overall and by respondent characteristics. Average acceptance of the COVID-19 vaccine across studies and subgroups within studies. For each study, we summarize sampling information in parentheses in the following way: (1) we indicate whether the geographic coverage of the sample is national or subnational. If the coverage is subnational we provide further details; (2) we list the number of observations included in the study. In the plot, points represent the estimated percentage of individuals who would take the vaccine. ‘No’, ‘Don’t know’ and ‘Refuse’ are taken as a single reference category. Bars around each point indicate a 95% confidence interval for the estimate. The ‘All LMICs (national samples)’ row reports averages for just the LMIC samples with national-level geographic coverage. An estimate of average acceptance for all studies in LMICs (excluding the United States and Russia) is also shown in the ‘All LMICs’ row.

Our main results are shown in Fig. 1 and are reproduced as Supplementary Table 1. The first column provides overall acceptance rates in each study, while the remaining columns disaggregate acceptance by respondent characteristics. The ‘All LMICs’ row reports averages for the LMIC samples included in our study and excludes Russia and the United States. The ‘All LMICs (national samples)’ row reports averages for just the LMIC samples with national-level geographic coverage.

The average acceptance rate across the full set of LMIC studies is 80.3% (95% confidence interval (CI) 74.9–85.6%), with a median of 78%, range of 30.1 percentage points (pp) and interquartile range of 9.7 pp. Our estimate of the between-study standard deviation *τ*, using a random effects meta-analysis model, is 0.084, which represents only 10.5% of our estimate of the average acceptance across LMIC studies.

The acceptance rate in every LMIC sample is higher than in the United States (64.6%, CI 61.8–67.3%) and Russia (30.4%, CI 29.1–31.7%). Reported acceptance is lowest in Burkina Faso (66.5%, CI 63.5–69.5%) and Pakistan (survey 2; 66.5%, CI 64.1–68.9%). Pakistan’s relatively low acceptance rate could be linked to negative historical experiences with foreign-led vaccination campaigns^22,25,26^. This hesitancy may be particularly problematic given the magnitude of the second wave in neighboring India and the acceleration of cases across South Asia that threatens to overwhelm health infrastructure. The relatively low acceptance rate in Burkina Faso might reflect general vaccine hesitancy. As shown in Table 2, fewer people believe that vaccines in general are safe in Burkina Faso than in any other country included in our study, except Russia.

We find limited evidence of variation across demographic subgroups in our aggregate analysis of LMIC samples, as shown in Supplementary Table 2. Women are generally less willing to accept the vaccine than men (average difference about 4.2 points, significant at *P* *<* 0.01). Respondents under age 25 years and less-educated respondents are marginally more willing to take the vaccine than older and more educated respondents, respectively, but these differences are not statistically significant.

Supplementary Table 3 provides results disaggregated by demographic subgroups for individual studies. The average gender differences in the aggregate LMIC analysis are driven by the Burkina Faso, Mozambique, Pakistan (survey 1), Rwanda and Sierra Leone (survey 1) samples. However, these gender differences in acceptance are less than 10 percentage points in each of these samples, in contrast to the larger gender gaps in acceptance we observe in the United States (17%) and Russia (16%).

Less-educated respondents expressed significantly higher acceptance in the Burkina Faso, Rwanda, Sierra Leone (survey 1) and Uganda (survey 2) samples, which represent the majority of studies from Sub-Saharan Africa. Notably, we observe the opposite pattern in the India, Pakistan survey 1 and Pakistan survey 2 samples. In all three of these studies, acceptance is greater among more educated respondents, although this difference is not statistically significant in the India sample. Education is also a positive and significant predictor of acceptance in the United States.

We find mixed evidence across studies with respect to the relationship between age and COVID-19 vaccine acceptance. In India and Nigeria, respondents younger than 25 years of age are significantly less willing to take the vaccine relative to adults who are 25–54 years old, while in Mozambique, Pakistan survey 1 and Rwanda, those under 25 years are significantly more willing. In Mozambique and Rwanda, respondents under 25 years are also significantly more accepting compared to those 55 years and over; however, the difference between these age groups is not statistically significant in other LMIC samples. In the United States and Russia, older respondents are consistently more accepting than younger respondents.

To better understand the reasoning behind vaccine acceptance, we asked those who were willing to take the vaccine why they would take it. We summarize these results in Supplementary Table 4, with additional details in Supplementary Table 5. The reason most commonly given for vaccine acceptance across samples is personal protection against COVID-19 infection. The average across the LMIC samples is 91% (CI 86–96%), with a median of 92.5% and a range of 22 pp. In every individual study, including the United States (94%, CI 92–95%) and Russia (76%, CI 74–78%), this ranks as the most cited reason. In distant second place in the aggregate LMIC analysis is family protection, with an average of 36% (CI 28–43%), a median of 34.5% and a range of 39 pp. In comparison to protecting oneself and one’s family, protecting one’s community does not feature prominently among stated reasons for acceptance. These reasons do not vary substantially by age group, as shown in Supplementary Table 6.

**Fig. 2:**
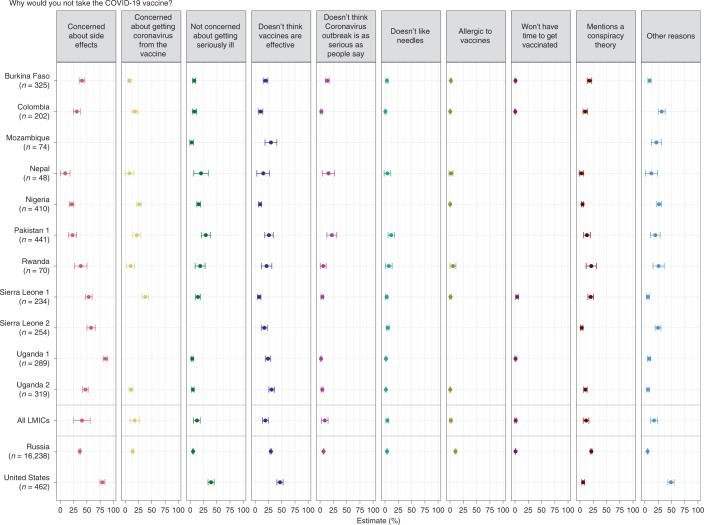
Reasons not to take the vaccine. The percentage of respondents mentioning reasons why they would not take the COVID-19 vaccine. In the plot, points represent the estimated percentage of individuals that would not take the vaccine or do not know if they would take the vaccine for each possible response option. Bars around each point indicate the 95% CI for the estimate. An estimated average for all studies in LMICs is also shown. The size of the points illustrates the number of observations in each response option. The India and Pakistan survey 2 studies are not included because they either did not include the question or were not properly harmonized with the other studies.

Figure 2 summarizes the reasons given by respondents who said they were not willing to take a COVID-19 vaccine. The results from Fig. 2 are reproduced in Supplementary Table 7. Concern about side effects is the most frequently expressed reason for reluctance in our LMIC samples. This concern is particularly evident among samples from Sub-Saharan Africa. In Uganda survey 1 (85.1%, CI 80.7–89.6%), Sierra Leone survey 2 (57.9%, CI 50.1–65.7%), Sierra Leone survey 1 (53.5%, CI 47.1–59.9%) and Uganda survey 2 (47.3%, CI 42.2–52.5%), a sizable percentage of respondents unwilling to take the vaccine cited worries about side effects. Respondents in Russia (36.8%, CI 35.2–38.4%) and even more in the United States (79.3%, CI 74.6–84%) frequently report this concern.

The Uganda survey 2 (31%, CI 25.9–36.2%), Mozambique (29.7%, CI 18.6–40.8%) and Pakistan survey 1 (26%, CI 18–34%) study samples show relatively high levels of skepticism about vaccine effectiveness among hesitant respondents. This is also true in Russia (29.6%, CI 28.1–31.1%) and the United States (46.8%, CI 41–52.6%). In addition, some hesitant respondents cite lack of concern about COVID-19 infection as a reason not to be vaccinated. This answer is particularly common in the United States (39.3%, CI 33.5–45%), Pakistan survey 1 (29.4%, CI 20.9–37.9%) and Nepal (20.4%, CI 6.7–34.1%) studies.

**Fig. 3:**
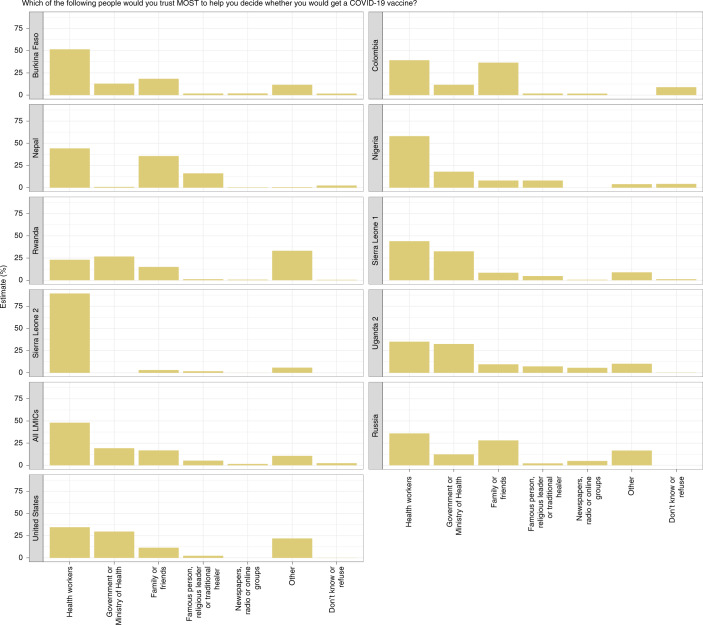
Trusted sources respondents say they would trust most to help them decide whether to take the COVID-19 vaccine. Histograms of sources respondents say they would trust most to help them decide whether to take the COVID-19 vaccine. Respondents were only permitted to select one most trusted actor or institution. The India, Mozambique, Pakistan survey 1, Pakistan survey 2 and Uganda survey 1 studies are not included because they either did not include the question or were not properly harmonized with the other studies.

In Fig. 3 we report respondents’ most trusted source of guidance when deciding whether to take a COVID-19 vaccine. The results from Fig. 3 are reproduced in Supplementary Table 8.

We find striking consistency across studies. In all samples except Rwanda, including those from Russia and the United States, respondents identify the health system as the most trustworthy source to help them decide whether to take the COVID-19 vaccine. The average across LMIC samples is 48.1% (CI 31.6–64.5%), with a median of 44.1% and range of 66.3 pp. Respondents in Sierra Leone survey 2 (89.3%, CI 87.2–91.5%), Nigeria (58%, CI 55.7–60.2%) and Burkina Faso (51.6%, CI 48.5– 54.8%) cited health workers most often. Sierra Leone has the highest trust in health workers and the Ministry of Health, potentially reflecting investments in public health following the 2014–2015 Ebola epidemic^27^.

**Extended Data Fig. 1:**
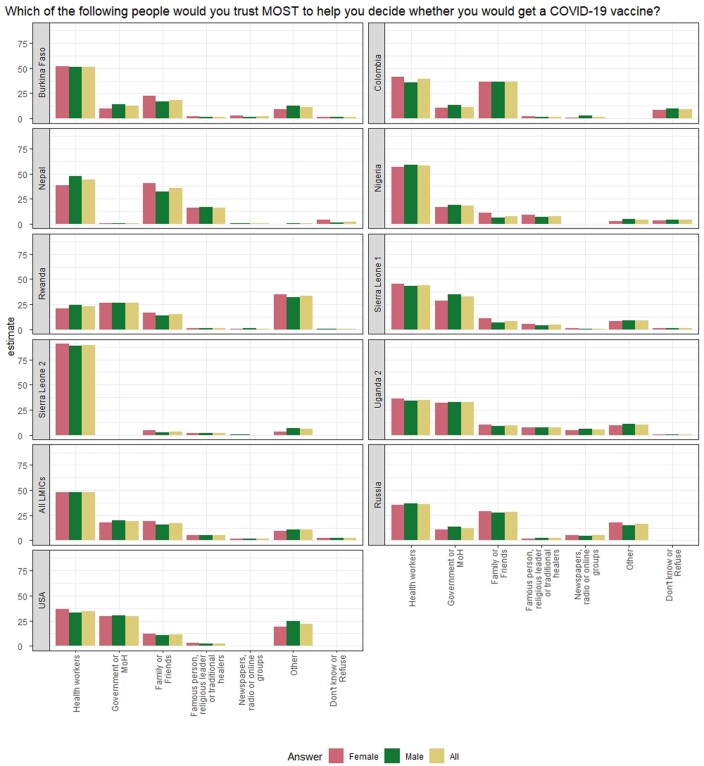
Trusted sources and institutions, broken down by gender. Figure ED1 shows histograms of sources and institutions that respondents say they would trust most to help them decide whether or not to take the COVID-19 vaccine. Respondents were only permitted to select one most trusted source or institution. Responses are broken down by gender of respondent.

In Colombia (36.6%, CI 33.5–39.7%), Nepal (35.6%, CI 32.9–38.3%), Russia (28.1%, CI 26.8–29.3%) and Burkina Faso (18.4%, CI 16–20.9%), the next most cited sources are family and friends. Across the pooled samples, women appear to be three percentage points more likely to rely on family and friends than male respondents, though this difference is not statistically significant (Extended Data Fig. 1).

By contrast, in Sierra Leone survey 1 (32.5%, CI 29.7–35.4%), Uganda survey 2 (32.4%, CI 29.9–35%), United States (29.7%, CI 27–32.3%) and Nigeria (18%, CI 16.2–19.8%), the government is the second most frequently cited. Religious leaders and celebrities are not seen as the top sources of guidance by many respondents in any sample other than Nepal, where many respondents say they most trust famous people (16.1%, CI 13.3–18.9%).

Finally, we highlight two idiosyncratic, yet frequently mentioned, trusted sources of information in deciding whether to take a COVID-19 vaccine. In Rwanda, 34% of respondents would most trust ‘themselves’ for guidance, the most frequent response in this sample. In the United States, 14% of respondents cited Joe Biden, then president-elect and therefore excluded from the ‘government’ category, as their most trusted source.

## Discussion

The current study contributes to the emerging picture of global vaccine acceptance by focusing on COVID-19 vaccine attitudes in a set of low-income and lower-middle-income countries. Our findings show variable but broadly high levels of prospective COVID-19 vaccine acceptance across the LMICs we study, using data from 20,176 respondents in 13 studies in 10 LMICs in Africa, South Asia and Latin America. Acceptance across these LMIC samples averaged 80.3%, ranging between 66.5% and 96.6% with a median of 78%. The two benchmark countries, Russia and the United States, demonstrate lower COVID-19 vaccine acceptance, consistent with lower pre-pandemic childhood vaccine confidence in these countries, as shown in Table 2.

Many metrics and indices measure vaccine acceptance and hesitancy globally^28–31^. Our surveys use measures employed in other COVID-19 vaccine acceptance studies^3,6–11^ and that are recommended by the WHO Data for Action guidance^32^, allowing for meaningful cross-study and cross-country comparisons. We measure trust in sources of information about COVID-19 vaccination using a measure similar to that used in the Vaccine Confidence Index (VCI), a widely used survey tool^4^.

Consistent with other studies, we find higher average vaccine acceptance among men than women^3,7–10^. In contrast to studies focused primarily on higher-income countries, we find no consistently significant differences with respect to age^7,9^ or education in our LMIC samples.

A key contribution of our study relative to the existing literature is its focus on the reasons why respondents express intentions to take (or refuse) a COVID-19 vaccine. Other work has highlighted appeals to altruistic behavior or other prosocial motivations to promote vaccine acceptance^33^, yet we find that the potential risks and benefits to personal well-being feature much more prominently in our respondents’ reasoning, suggesting that appeals about personal protection could be more effective in the countries under study here.

The most commonly stated reason for vaccine refusal is concern about safety (side effects). The vast majority (86%) of our surveys were conducted as reports from phase 2 and 3 clinical trial data were emerging for the earliest commercially available vaccines, but prior to the first Emergency Use Authorization of any vaccine (the BNT162b vaccine was approved by the United States on 11 December 2020). Early trial data showed that severe adverse effects were extremely rare^34–39^, occurring in fewer than 10% of people in clinical trials^40^. Our respondents’ concern about side effects could reflect the rapid pace of vaccine development^41^ and the limited information available about potential COVID-19 vaccine safety at the time of data collection. These concerns could also reflect worries about mild, yet common and transient side effects, such as fatigue, muscle pain, joint pain and headache.

Intensive media coverage of adverse events may exacerbate concerns about side effects^42^. In particular, new information about rare but severe cases of thrombosis associated with the AstraZeneca vaccine that appeared after our data collection period could affect hesitancy levels. This is of particular relevance to LMICs, which are likely to rely on the AstraZeneca vaccine in their immunization campaigns through initiatives such as COVAX.

Concerns about vaccine efficacy, averaging 19.2% in the LMIC samples, may also reflect a lack of information about vaccines at the time of our surveys. However, we note that respondents in our samples rarely cited conspiracy theories about ulterior motives on the part of corporations, politicians or the pharmaceutical industry, despite attention given to fears about these issues in higher-income countries^43^.

Our study has several limitations, which we address here. First, our data are not representative of all LMICs. They instead represent a sample of studies in countries where our organizations could quickly and safely mobilize coordinated data collection. Second, samples from the countries we include here are not fully nationally representative. Phone surveys, although necessary during a global pandemic, do not include individuals who reside outside coverage areas, lack access to a cell phone or do not respond to calls. In addition, as shown in Table 3, several studies focus on subnational populations of interest from pre-existing studies to which questions about COVID-19 vaccination were added. Particular care should be taken in extrapolating these findings to national populations.

**Extended Data Fig. 2:**
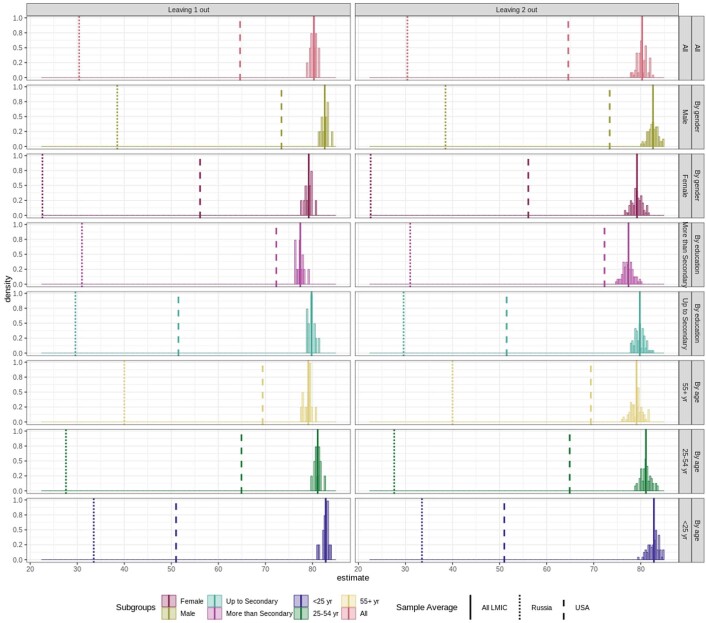
Average vaccine acceptance across all LMIC countries leaving one or two study samples out. Figure ED2 shows distribution of estimates of average acceptance for all studies in LMICs (excluding USA and Russia) leaving one and two study samples out at a time. Figure also shows distributions of subgroup averages by gender, education and age leaving one and two study samples out at a time. To directly compare the resulting distributions to the estimates reported in Fig. 1, we plot point estimates reported in Fig. 1 for all LMIC studies, Russia and the US.

In spite of this variation in sample composition, our main findings—of high COVID-19 vaccine acceptance in our LMIC samples relative to the United States and Russia—are remarkably consistent across studies. We conduct several robustness checks to probe the sensitivity of our aggregate LMIC analysis to the inclusion of particular samples. First, as shown in Extended Data Fig. 2, we re-estimate aggregate vaccine acceptance across our LMIC samples, successively dropping one and two study samples at a time. Regardless of which samples are excluded, the average vaccine acceptance rate among LMIC samples remains consistent and considerably higher than in the United States and Russia, demonstrating that our results are not driven by the peculiarities of one or two studies. Second, we repeat the same analysis excluding all samples that are subnational in scope, which yields a mean acceptance rate of 78.4% (CI 67.9–89%), as shown in the row ‘All LMICs (national samples)’ in Fig. 1 and Supplementary Table 1.

The expressed intentions to take a COVID-19 vaccine that we document in our LMIC samples, if translated into behavior, would meet or exceed the current herd immunity threshold for COVID-19 in many, but not all, countries (estimated to be between 70 and 80%, based on the predominant variant in circulation in different countries)^44–46^. Reported intentions may, however, not always translate into vaccine uptake^47^. The high salience of COVID-19 may have increased reported intentions. Conversely, reports about side effects and risks associated with expedited vaccine development may have increased hesitancy. The fast-moving pandemic and vaccine development context may change perceptions about vaccines by the time they are widely available in LMICs.

Indeed, previous research on vaccine hesitancy has emphasized how concerns that arise surrounding vaccination campaigns are often case- and context-specific^48^, making it difficult to predict exactly how COVID-19 vaccines will be received in any given setting. The lower COVID-19 vaccine acceptance rates we observe in Russia and the United States, for example, may reflect the politicization of this specific pandemic and of vaccine development^49–52^, in addition to generally greater vaccine skepticism.

Nonetheless, our findings suggest several concrete implications for policy relating to vaccine rollout in LMICs. First and foremost, we document high levels of COVID-19 vaccine acceptance in our LMIC samples compared to Russia and the United States. Although global vaccine distribution has skewed heavily toward higher-income countries so far^3^, our findings suggest that prioritizing distribution to LMICs is justified not only on equity grounds, but on the expectation of higher marginal returns in maximizing global coverage more quickly.

The high stated acceptance rates we document also imply that, once vaccine distribution to LMICs begins in earnest, interventions should focus on converting positive intentions into action. Straightforward, low-cost nudges may be effective in this regard. Two recent large-scale studies in the United States found that vaccination appointment reminder messages from healthcare providers increased influenza vaccine uptake^53,54^. Similar interventions have proven effective in increasing immunization in LMIC contexts. In Ghana and Kenya, vaccination reminders combined with small cash incentives increased childhood immunization coverage^55,56^. Cash and in-kind incentives programs were also effective in Nigeria and India^57,58^.

This recommendation is consistent with accepted frameworks, such as the WHO’s Behavioral and Social Drivers of vaccination (BeSD) model, which suggest leveraging favorable intentions through reminders and primes, and reducing access barriers when the vast majority of people intend to be vaccinated^32,59^. In particular, because COVID-19 vaccination may be more collectively than individually optimal, ease of access is critical to achieving high coverage^60^. The availability of single-dose vaccines could be advantageous in settings with high vaccination demand but relatively low-capacity healthcare systems, as is the case in many LMICs.

Our findings also suggest directions for the design and delivery of messaging to address remaining COVID-19 vaccine hesitancy in the countries under study here. Persuasion campaigns may be particularly important in countries where acceptance rates are below herd immunity thresholds. We highlight three potential implications for messaging below, but urge policymakers and stakeholders to utilize country-specific results to develop further strategies that may work best in their particular context. We also echo calls for integrating rigorous impact evaluation of vaccine hesitancy interventions in all contexts, given limited evidence so far^61^.

First, our data strongly support the view that respondents from the included set of LMICs prefer to follow the guidance of practitioners with the most relevant knowledge and expertise. We find high levels of trust in health workers, which suggests that social and behavioral change communication strategies engaging local health workers may be particularly effective in combating remaining hesitancy^50,62^. Health workers have also been the first group to receive the COVID-19 vaccine and are therefore best positioned to share locally credible experiences of vaccination^63^. Although celebrities were rarely identified as a most trustworthy source for COVID-19 advice in our study, celebrity endorsements have proven effective in other contexts and may complement a strategy that primarily focuses on health workers^64^.

Second, our findings offer some guidance on the specific content of vaccine messaging that is likely to be most effective in persuading those who may be hesitant. Hesitant respondents were most concerned about side effects and vaccine efficacy. This suggests that proactive messaging, initiated before large-scale vaccination campaign rollout, should highlight the high efficacy rates of the COVID-19 vaccines currently on the market in reducing or eliminating disease, hospitalizations and death, and communicate accurate information about potential side effects, including the rarity of severe adverse events that may have contributed to hesitancy through widespread media coverage^65,66^.

Third, consistent with previous studies on COVID-19 vaccination^3,7–10^, our study finds lower vaccine acceptance, on average, among women than men, suggesting that messaging strategies focusing on women may be important in addressing overall hesitancy. Recent work in Latin America on COVID-19 vaccine messaging found that the provision of basic information about the vaccines was particularly effective in persuading hesitant women^67^. More generally, countries may consider tailoring their messaging campaigns to address concerns held by more hesitant subpopulations, which vary across our samples with respect to age, gender and education. Additional research is needed to identify and design effective messaging campaigns to overcome hesitancy among specific subpopulations in each setting^61,65^.

Finally, the high coverage rates of existing vaccines, coupled with respondents’ reliance on friends and family as information sources, suggest that the general pro-vaccination stance of many LMIC citizens could be leveraged to increase uptake of COVID-19 vaccines as they become available. This might yield particularly strong results in Colombia and Nepal, where family members and friends are seen as an important source of advice when deciding whether to take a COVID-19 vaccine. Social learning strategies and norm-setting are powerful drivers of behavior in many related sectors. Social signaling of positive attitudes towards vaccines may help shift social norms toward even greater immunization acceptance and uptake in the community at large^68–70^. As with messaging, policymakers should consider designing and evaluating social mobilization strategies targeted toward more hesitant subgroups^71^.

## Methods

### Study protocols

All studies analyzed in this Article were conducted via phone or online (United States and Russia) between 17 June 2020 and 18 January 2021. Table 3 presents the geographic scope, sampling methodology and survey modality for each study.

### Consent

All participants consented to participation in the research either verbally or via online forms (United States and Russia only).

### Ethical approvals

Each of the individual studies independently obtained IRB approval. The Burkina Faso study was approved via IPA IRB protocol 15608 and the Burkina Faso Institutional Ethics Committee for Health Sciences Research approval A13-2020. The Colombia study was approved via IPA IRB protocol 15582. The India study was approved via the London School of Economics (REC ref. 1132). The Mozambique study was approved by Universidade Nova de Lisboa. The Nepal study was approved via Yale University IRB protocol 2000025621. The Nigeria study was approved via the IRB at the University of Pennsylvania (protocol 834548). Pakistan survey 1 was approved via Princeton University IRB protocol 7250. Pakistan survey 2 was approved via Lahore University of Management Sciences IRB protocol LUMS-IRB/07012020SA. The Rwanda Research for Effective COVID-19 Responses (RECOVR) study was approved via IPA IRB protocol 15591, Rwanda National Institute for Scientific Research permit no. 0856/2020/10/NISR and Rwanda National Ethics Committee approval no. 16/RNEC/2020. The Russia study was approved via Columbia University IRB protocol IRB-AAAT4453. Sierra Leone survey 1 was approved via IPA IRB protocol 15592 and the Sierra Leone Ethics and Scientific Review Committee (no number provided, letter available upon request), and Sierra Leone survey 2 was approved via the Sierra Leone Ethics and Scientific Review Committee (SLERC 2904202) and Wageningen University (24062020). Uganda survey 1 was approved via Mildmay Uganda Research Ethics Committee (protocol no. 0109-2015). Uganda survey 2 was approved via IPA IRB protocol no. 15018, WZB Berlin Social Science Center Ethics Review Board protocol no. 2020/0/91, NYU Abu Dhabi IRB protocol no. HRPP-2020-64, MIT Committee on the Use of Humans as Experimental Subjects protocol no. 2005000155 and the Mildmay Uganda Research Ethics Committee protocol no. 0604-2019. The US study was approved via Cornell University IRB under protocol no. 2004009569.

### Survey questions and sample construction

Survey data were collected between June 2020 and January 2021. Our main outcome measure was vaccine acceptance. Across studies, we asked respondents, ‘If a COVID-19 vaccine becomes available in [your country], would you take it?’. This measure aligns with widely reported COVID-19 vaccine acceptance measures^3,6–11^. If the respondent answered yes to this question, we followed up with the question, ‘Why would you take it? [the COVID-19 vaccine]’. If the respondent said they would not be willing to take the vaccine, we followed up with the question, ‘Why would you not take it? [the COVID-19 vaccine]’. Finally, regardless of their expressed willingness to take the vaccine, we asked about groups and institutions that would be most influential in their decision: ‘Which of the following people would you trust MOST to help you decide whether you would get a COVID-19 vaccine, if one becomes available?’ following recent work on vaccine confidence^4^. To examine heterogeneity across demographic strata, we collected information about gender, age and education. Slight variations in question wording and answer options across studies are documented in Supplementary Tables 9–12.

The studies varied in terms of geographic scope, sampling methodology and survey modality. Seven were national or nearly national in scope. Studies from Burkina Faso, Colombia, Rwanda and Sierra Leone (survey 1) used nationally representative samples of active mobile phone numbers reached through random digit dialing (RDD). Sierra Leone survey 2 was conducted by phone among a random sample of households in 195 rural towns across all 14 districts of the country. Studies in the United States and Russia were conducted online using quota samples obtained from private survey companies.

The remaining eight studies targeted subnational populations. One study from Pakistan (Pakistan survey 2) used RDD in Punjab Province. Respondents in Mozambique, Nigeria, Pakistan (Pakistan survey 1), Uganda (Uganda survey 1 and Uganda survey 2), India and Nepal were drawn from pre-existing studies to which COVID-19 vaccine questions were subsequently added. For example, Uganda survey 1 sampled female caregivers of households in rural and semi-rural villages as part of a large ongoing cluster randomized controlled trial implemented across 13 districts.

Table 3 summarizes the dates of data collection, geographic scope, sampling methodologies and survey modalities of all 15 studies.

All surveys were conducted remotely to minimize in-person contact and comply with social distancing guidelines. Interviews were conducted by local staff in each country in the local language(s). Surveying by phone made rapid, large-scale data collection possible. In two samples, the United States and Russia, surveys were carried out via online polling. All surveys lasted ~15–40 min.

Taken together, we have data from 20,176 individuals from 10 LMICs and 24,084 from the United States and Russia, for a total of 44,260 respondents. We report data missingness patterns in Supplementary Table 13.

### Detailed study protocols

The Burkina Faso, Colombia, Rwanda and Sierra Leone survey 1 samples were drawn from the RECOVR studies implemented by IPA. The target population for these studies comprised all adults with mobile phone numbers in the country, based on national communications authority number allocation plans. The sampling frame consisted of all mobile phone numbers in the countries. Numbers were called via RDD, stratified by mobile network operator market share for a two-round panel survey. In Burkina Faso, the sample included 977 respondents contacted in the second round of a panel of 1,383. In Colombia, the sample included 1,012 respondents contacted in the second round of a panel of 1,507. In Rwanda, the sample included 1,355 respondents contacted in the second round of a panel of 1,480. In Sierra Leone survey 1, the sample included 1,070 respondents contacted in the second round of a panel of 1,304. Post-stratification weights were computed to adjust for differential attrition between the first and second rounds of the RDD panel, weighting on gender, region and educational attainment.

The ‘India, Coping with COVID-19 in Slums’ subnational sample was drawn from research undertaken by the Nova School of Business and Economics, The Institute for Fiscal Studies, and the University of St Andrews. The target population was a random subset of slum populations in Lucknow and Kanpur, Uttar Pradesh, India. Socio-economic variables were only collected for a representative sample of the population relying on community toilets or open defecation to fulfill their sanitation needs. The study design was a randomized controlled trial, with complete census of households within 142 slums (carried out from September to December 2017) and a series of household and caretaker surveys, objective measurements, incentivized behavioral measurements and a structured community activity, collected for a subset of 100 slums between April 2018 and September 2019. The catchment areas of community toilets were randomly allocated to two interventions. The first intervention aimed at community toilet improvements by offering caretakers the choice of a grant to be spent for improvements in the facility. Following the grant, caretakers were offered a large financial reward conditional on the cleanliness of the facility. The second intervention added to this community toilet improvement awareness creation through face-to-face information sessions, leaflets, monthly reminders using voice messages sent to mobile phones, and posters hung in the community toilets. A two-step sampling was applied: study households from the main study sample were first sampled, then households from the whole slum population were added. The baseline ran from June to July 2020, follow-up 1 ran from October to November 2020, and follow-up 2 ran from 16 December 2020 to 18 January 2021. The sample size was 3,991 households, with a mean of 28 households per cluster (142). Baseline non-response was 25%, and the attrition rate from baseline to follow-up (1 and 2) was 13%. The study included 1,277 randomly selected replacement households for follow-up (1 and 2). Sampling weights are included.

The Mozambique subnational sample, implemented by the International Growth Center and the Nova School of Business and Economics, targeted microentrepreneurs in the urban markets of Maputo and household heads from the province of Cabo Delgado. The initial data were collected in person in two different studies. For microentrepreneurs in Maputo, the data were collected between October 2013 and April 2014 (baseline) and between July and November 2015 (endline). For household heads in Cabo Delgado, the data were collected in person between August and September 2016 (baseline) and between August and September 2017 (endline). The first study was dedicated to analyzing the impacts of interventions targeting microentrepreneurs in urban markets on financial inclusion and literacy. The second study focused on the role of information to counteract the political resource curse after a substantial natural gas discovery. The first initial sample was selected by in-field random sampling in 23 urban and peri-urban markets in Maputo and Matola. Stratification was based on the gender of the respondent and on the type of establishment (stall versus store). The second initial sample was selected to be representative of 206 communities in the province of Cabo Delgado, randomly drawn from the list of all 421 polling locations in the sampling frame, stratified on urban, semi-urban and rural areas. The sample includes 554 microentrepreneurs from Maputo and 308 households from Cabo Delgado.

The Nepal Western Terai Panel Survey (WTPS) subnational sample was implemented by researchers from Yale University and the Yale Research Initiative on Innovation and Scale (Y-RISE). Its target population was rural households in the districts of Kailali and Kanchanpur. Initial baseline data was collected in person in July 2019, and five rounds of phone survey data were collected between 12 August 2019 and 4 January 2020. The phone survey sample included 2,636 rural households in the districts of Kailali and Kanchanpur that represent the set of households that responded to phone surveys from an original sample of 2,935 households. This sample was constructed by randomly sampling 33 wards from 15 of the 20 subdistricts in Kailali and Kanchanpur and selecting a random 97 villages from within those wards. At the time of baseline data collection in July 2019, 7 of these 97 villages were dropped from the sample due to flooding. Households belong to the bottom half of the wealth distribution in these villages, as estimated by a participatory wealth-ranking exercise with members of the village. The sample included in this study comprised 1,392 households.

The Nigeria subnational sample was implemented by researchers from WZB Berlin Social Science Center and the University of Illinois Chicago. The target population included Christian and Muslim men and women, aged 18 years and above, living in Kaduna State, Nigeria. Initial data were collected from a subset of the sample in December 2019 (in person survey) and July to August 2020 (phone survey) as part of an experiment testing the effects of a brief radio program on inter-religious animus. A random walk procedure and random sampling were used within households to recruit a representative sample of adults in Kaduna town. The rest of the sample was recruited for the study in August 2020 by purchasing phone lists of residents of Kaduna State. The subset of the sample in the radio study was randomly assigned to listen to a brief radio program on one of the following topics: (1) an inter-religious storyline, (2) an intra-religious storyline and (3) a message about maintaining safe health practices. All respondents in the sample participated in a study examining the effect of viewing an inter-religious storyline unfolding over a full season of a popular TV drama, Dadin Kowa. The season aired from August to October 2020. A third of the sample were encouraged to watch Dadin Kowa, a third were encouraged to watch the TV station Africa Magic Hausa at the same time Dadin Kowa aired, and a third were in the treatment-as-usual group. All participants received a weekly incentivized SMS quiz from August to October 2020. The survey from which the data were drawn is not primarily about COVID-19, but was designed as an endline survey to follow the TV drama intervention described above. The goal of the COVID-19 survey was to measure a range of attitudinal outcomes related to Christian–Muslim relations (including prejudice, intergroup threat perceptions, dehumanization and support for the use of violence, among others). We included nine of the standardized COVID-19 vaccine-related questions collected specifically for this vaccine acceptance study in the final module of the endline survey. A total of 950 respondents in the sample were recruited in person through a random sampling procedure in the Kaduna metropolitan area (pre-COVID). The remaining 1,700 respondents were recruited into the study over the phone from lists of phone numbers of Kaduna State residents that were purchased from a private vendor. All 1,834 individuals who completed the endline survey were included.

The Pakistan survey 1—the Sheikhupura Police Study Sample—was implemented by the Institute of Development and Economic Alternatives, Lahore University of Management Science, London School of Economics and Princeton University. The target population was a representative sample of adults from 108 of 151 police beats in Sheikhupura and Nankana districts of Punjab Province. The survey involved calls to all households in the stratified random sample for the policing study midline survey. The sampling frame included households in Sheikhupura and Nankana districts, and the *s*ample comprised 1,473 respondents. Post-stratification weights were computed to adjust for the sampling process, which involved stratifying first on 27 police stations, then within each police station on beats, then probability proportional to size (PPS) sampling within beats using Asiapop population data.

The Pakistan Economic Vulnerability Assessment (EVA) subnational sample (Pakistan survey 2) was implemented by Lahore University of Management Studies and targeted all possible mobile phone numbers in the province of Punjab generated based on the local mobile phone number structure in Pakistan. The survey involved making calls to individuals in Punjab based on RDD. The sample included 1,492 respondents.

The Russian Federation, Research on COVID-19 in Russia’s Regions (RoCiRR) subnational sample was implemented by the International Center for the Study of Institutions and Development (HSE University, Moscow, Russia) and the Economics Department of Ghent University and Columbia University. The target population was adult internet users who resided in one of 61 federal subjects (federal cities, oblasts, republics, krais and autonomous okrug) of Russia. The regions included in the study were the following republics (Bashkortostan, Karelia, Komi, Mariy El, Mordovia, Tatarstan, Udmurtia, Chuvashia), krais (Altai, Krasnodarsky, Krasnoyarsky, Permsky, Primorsky, Stavropolsky, Khabarovsky), oblasts (Arkhangelsk, Astrakhan, Belgorod, Bryansk, Vladimir, Volgograd, Vologda, Voronezh, Ivanovo, Irkutsk, Kaliningrad, Kaluga, Kemerovo, Kirov, Kostroma, Kurgan, Kursk, Leningrad, Lipetsk, Moscow, Murmansk, Nizhny Novgorod, Novgorod, Novosibirsk, Omsk, Orenburg, Orel, Pskov, Penza, Rostov, Ryazan, Samara, Saratov, Sverdlovsk, Smolensk, Tambov, Tver, Tomsk, Tula, Tyumen, Ulyanovsk, Chelyabinsk, Yaroslavl), as well as Moscow, Saint Petersburg and Khanty-Mansiysk Autonomous Okrug—Ugra. The remaining 24 federal subjects were excluded from the study due to the inability to enroll a sufficient sample size with the desired characteristics (sample size, age, gender and education group composition) and because they accounted for less than 14% of the total adult population of Russia. The study was designed to measure the impact of pandemics on Russians, mostly those who live in cities with more than 100,000 residents. It contained a number of questions on personal experience, norms and values, trust in government institutions, provision of social services and mass media use. The region and geolocality of every respondent were recorded. In total, 25,558 respondents received the module on vaccine acceptance. The sample was enrolled from the pool of Russian online survey company OMI (Online Market Intelligence). The sampling was specifically targeted at having a minimum of 150 respondents in each of the 61 regions, including respondents from all the main age and gender groups within each region. Respondents were also selected so that at least 40% of respondents did not have higher education, in accordance with higher education rates in Russia. Of 25,558 recruited respondents, 22,125 completed the survey. Among the 22,125 respondents who completed the survey, 20,821 were enrolled from the general pull of the survey company respondents, while the remaining 1,304 respondents were enrolled among residents of cities with populations below 100,000 and rural areas. Post-stratification weights were computed to match marginal distributions of age, gender and education among the adult population of Russia with target proportions coming from the 2019 Yearbook and 2015 Microcensus released by the Russian Federal Bureau of National Statistics (Rosstat).

The Sierra Leone Rural Electrification (SLRE) project sample (Sierra Leone survey 2) included towns that were candidates for rural electrification. This nationwide sample was implemented by the International Growth Centre (IGC), Wageningen University & Research, Yale Research Initiative on Innovation and Scale (Y-RISE), WZB Berlin Social Science Center and Columbia University. The study included households in 195 rural towns across all 14 districts of Sierra Leone. Of these, 97 villages were selected to benefit from an electrification program. For the original study, initial baseline data were collected during late 2019 and early 2020 as part of a study to assess the impact of rural electrification in rural towns in Sierra Leone. The Government of Sierra Leone (GoSL) in collaboration with the United Nations Office for Project Services (UNOPS) and international donors was implementing the Rural Renewable Energy Project (RREP). In its first wave, during 2017, the project provided stand-alone solar photovoltaic powered mini-grids to 54 communities across the country. Construction of mini-grids in 43 additional towns is ongoing. In RREP communities, engineers construct 6- to 36-kW power mini-grids that provide reliable power year-round. Electricity is free for schools and clinics. Residential and commercial users can acquire connections from commercial operators. Household data were collected in 195 towns across all 12 districts of Sierra Leone. The GoSL selected 97 towns with (planned) mini-grids. We used propensity score matching to select 98 control communities. Within the communities, respondents were randomly selected from a census roster stratified by occupation status: farmers, business owners and other occupations (47%, 47% and 7%). In each village, the intended sample was 43 households (20 farmers, 20 businesses, 3 others). Data were collected during June to July (108 communities) and November to December 2019 (87 communities). If a household on the sampling list was not available on the village visit day, a randomly sampled list of replacement households were available to survey. The replacement household would be the same occupation as the sampled household would have been to maintain the sample ratio of 20-20-3 in each community. The goal of the COVID-19 survey was to assess households’ degree of economic vulnerability in the face of the COVID-19 pandemic. The COVID-19 survey data comprised 2,110 respondents from 186 towns from the original baseline survey. Phone surveys were attempted to all 195 rural communities from the baseline survey. The total baseline household sample comprised 7,047 respondents. We recontacted all baseline respondents that listed a phone number (4,594 respondents) and obtained informed consent for the phone survey. We implemented several waves of the phone survey, recontacting a respondent about every month. In wave 7, we added questions related to vaccine acceptability. Data collection took place between 7 October 2020 and 20 January 2021, with 2,110 respondents in 186 towns, for a tracking rate of 46%.

The Uganda survey 1 subnational sample was implemented by the International Growth Center, Trinity College Dublin, Stockholm School of Economics and Misum, Institute for International Economic Studies, Stockholm University. The target population was women from semi-rural and rural villages across 13 districts in Uganda (Iganga, Kayunga, Mbale, Mityana, Apac, Dokolo, Gulu, Adjumani, Koboko, Maracha, Nebbi, Soroti and Kumi). For the original study, initial baseline data were collected in 2016 as part of a large cluster randomized controlled trial, with the aim of selecting households likely to have children during the study period. Four criteria for selection were thus used (in descending order of importance): the household has a woman that is currently pregnant, is aged 16–30 years, has a young child less than 3 years of age, and/or is married (formally or informally). In each household, the respondent was chosen as the female household head or the primary female healthcare giver of the household if the household head could not be found. The COVID-19 survey data were collected through multiple rounds of phone surveys. The variable measuring age was constructed by approximation, using the baseline data from 2016 and adding four years to the 2016 measure. When the baseline respondent was replaced, the initial age information was deleted. Households were selected within 500 clusters (the village of the household). Out of 2,743 respondents, 1,752 were included, provided that they answered the main question about vaccine uptake.

The Uganda survey 2 subnational sample was implemented by WZB Berlin Social Science Center and Columbia University, NYU Abu Dhabi and IPA. The target population was all residents of Kampala who were Ugandan citizens, above the age of 18 years and agreed in principle to attend a short citizen consultative meeting. For the original study, baseline data were collected between July and October 2019 for an intervention that randomized citizen attendance to a set of 188 consultative meetings organized across Kampala. The meetings were organized to collect citizen preferences for the design of a forthcoming municipal citizen charter. The study also aimed to assess patterns of political inequality in meeting participation, dynamics and outcomes, as well as to study the subsequent effects on prosociality of being incorporated in this participatory process. One-third of the sample was randomly allocated as control and two-thirds of respondents were invited to attend a consultative meeting. The consultations took place between November 2019 and February 2020 across Kampala divisions. The intervention consisted of attendance at the consultative meeting organized a few months after baseline data collection. A further randomization allocated half of the invited participants to a meeting moderated by a local bureaucrat, while the remaining ones attended a meeting moderated by a neutral discussion leader. The COVID-19 survey sample comprised the 2,189 respondents to the baseline who were selected on the basis of their residence in the city. Having received permission to recontact these individuals, we coordinated a three-wave panel throughout the summer and fall of 2020, with respondents contacted via phone. The goal was to assess households’ degree of economic vulnerability in the face of the COVID-19 pandemic and respondents’ evaluations of the performance of political actors in tackling the pandemic. The 2,189 respondents to the baseline were randomly selected from a sampling frame of all buildings in Kampala for which information about their geographical coordinates was available. After randomly selecting a set of candidate structures, interviewers sampled respondents from the subset of structures that were residential. Of the 2,189 respondents that we aimed to contact, we were able to reach 1,333 in wave 1, 1,289 in wave 2 and 1,366 in wave 3. Wave 3 contained the COVID-19 vaccine module presented in this analysis.

The United States nationwide sample was implemented by WZB Berlin Social Science Center, Cornell University and Tufts University. The target population was a nationwide sample of adult internet users recruited through the market research firm Lucid. This survey was part of a panel study on attitudes toward COVID-19 technologies and public health surveillance. The Lucid Marketplace is an automated marketplace that connects researchers with willing online research participants. Lucid partners with a network of companies that maintain relationships with research participants by engaging them with research opportunities. Although Lucid does not provide probability samples of the US adult population, its quota samples approximate the marginal distributions of key demographic characteristics. Recent validation exercises have found that Lucid samples approximate nationally representative samples in terms of demographic characteristics and survey experiment effects^72^. The sample includes 1,959 individual online surveys. In the main question regarding intention to take the vaccine, ~10% of respondents (184) did not answer. Post-stratification weights were computed to match marginal population distributions of income, age, education, gender, race and region among the US adult population, with target proportions based on the 2018 American Community Survey.

### COVID-19 case history for countries included in the study

In the following we detail the COVID-19 case history for each country included in our study, based on data reported in the Johns Hopkins University Center for Systems Science and Engineering (JHU CSSE) database^73^.

In Burkina Faso, the first confirmed case of COVID-19 was recorded 9 March 2020. As of 15 October 2020 (the first date of the survey round containing the questions used in this study), 2,335 confirmed cases and 65 COVID-19 deaths had been reported in the country.

In Colombia, the first confirmed case of COVID-19 was recorded 6 March 2020. As of 15 August 2020 (the first date of the survey round containing the questions used in this study), 456,689 confirmed cases and 14,810 COVID-19 deaths had been reported in the country.

In India, the first confirmed case of COVID-19 was recorded 30 January 2020. As of 17 June 2020 (the first date of the survey round containing the questions used in this study), 366,946 confirmed cases and 12,237 COVID-19 deaths had been reported in the country.

In Mozambique, the first confirmed case of COVID-19 was recorded 22 March 2020. As of 30 October 2020 (the first date of the survey round containing the questions used in this study), 12,777 confirmed cases and 91 COVID-19 deaths had been reported in the country.

In Nepal, the first confirmed case of COVID-19 was recorded 23 January 2020. As of 1 December 2020 (the first date of the survey round containing the questions used in this study), 233,452 confirmed cases and 1,529 COVID-19 deaths had been reported in the country.

In Nigeria, the first confirmed case of COVID-19 was recorded 28 February 2020. As of 18 November 2020 (the first date of the survey round containing the questions used in this study), 65,693 confirmed cases and 1,163 COVID-19 deaths had been reported in the country.

In Pakistan, the first confirmed case of COVID-19 was recorded 26 February 2020. As of 24 July 2020 (the first date of the survey round containing the questions used in Pakistan survey 1), 271,887 confirmed cases and 5,787 COVID-19 deaths had been reported in the country. For Pakistan survey 2, by 2 September 2020, 297,014 confirmed cases and 6,328 COVID-19 deaths had been reported in the country.

In Russia, the first confirmed case of COVID-19 was recorded 31 January 2020. As of 6 November 2020 (the first date of the survey in this study), 1,720,063 confirmed cases and 29,654 COVID-19 deaths had been reported in the country.

In Rwanda, the first confirmed case of COVID-19 was recorded 14 March 2020. As of 22 October 2020 (the first date of the survey round containing the questions used in this study), 5,017 confirmed cases and 34 COVID-19 deaths had been reported in the country.

In Sierra Leone, the first confirmed case of COVID-19 was recorded 20 March 2020. As of 2 October 2020 (the first date of the Sierra Leone Study 1 survey round containing the questions used in this study), 2,252 confirmed cases and 72 COVID-19 deaths had been reported in the country. As of 7 October 2021 (the first date of the Sierra Leone Study 2 survey round containing the questions used in this study), 2,287 confirmed cases and 72 COVID-19 deaths had been reported in the country.

In Uganda, the first confirmed case of COVID-19 was recorded 21 March 2020. As of 21 September 2020 (the first date of the Uganda 1 survey round containing the questions used in this study), 6,468 confirmed cases and 63 COVID-19 deaths had been reported in the country. As of 23 November 2020 (the first date of the Uganda 2 survey round containing the questions used in this study), 18,165 confirmed cases and 181 COVID-19 deaths had been reported in the country.

In the United States, the first confirmed case of COVID-19 was recorded 20 January 2020. As of 4 December 2020 (the first date of the survey round containing the questions used in this study), 14,499,637 confirmed cases and 281,678 COVID-19 deaths had been reported in the country.

### Statistical analysis

Vaccine acceptance was defined as the percentage of respondents who answered ‘yes’ to the question, ‘If a COVID-19 vaccine becomes available in [country], would you take it?’. This was calculated combining all other answer options (‘No’, ‘Don’t know’ and ‘Refuse’) into a single reference category. We estimated the average acceptance for each individual sample via ordinary least squares (OLS) weighted by the respective study population weights and using robust standard errors clustered at the level of the sampling cluster.

In addition to study-level estimates, we combined data from all studies other than the United States and Russia to calculate an aggregate ‘All LMIC studies’ estimate. For these analyses, we estimated the average acceptance by OLS with weights for each study normalized such that the total weight given to observations was constant across studies. Robust standard errors for these analyses were clustered at the study level.

We note the core results would be virtually unchanged at 80.8% (74.5–87.1) rather than 80.3% (74.9–85.6) using countries rather than studies as groups in the pooled analysis, that is, if we set weights so that the sum of weights in each country (rather than in each study) sum to a constant and cluster standard errors at the country level (rather than the study level).

In this combined analysis, we also estimated the underlying heterogeneity of vaccine acceptance across studies using the between-studies variance estimator *τ*^2^ from a random effects model.

We conducted subgroup analyses by gender, age and education level and reported differences between groups. For age, we selected cutoffs of below 25 years, between 25 and 54 years, and 55 years and above, closely following the age breakdown proposed by recent work on COVID-19 vaccine acceptance^11^. However, the lower life expectancy (63 years on average)^74^ and younger-skewing populations (only 5% of the population is above 65 years old)^75^ of low-income countries, in particular, precluded further disaggregation at the upper end of the age distribution. For education, we divided the sample between respondents who had completed secondary school and those who had not. We defined these two groups to reflect broader schooling trends in LMICs, where out of every 100 students entering primary education, 61% complete lower secondary education^76^. The subgroup analyses estimates are calculated in exactly the same way as the overall acceptance rate—with weights again normalized to sum to a constant within each study—with the exception that the subsample used in the analysis is limited to those respondents fitting each demographic group.

We then investigated stated reasons for COVID-19 vaccine acceptance and hesitancy, and the types of sources respondents would trust most when making the decision about whether to take a COVID-19 vaccine. We report estimates of agreement with reasons for vaccine acceptance/hesitancy and trust in sources for individual studies and for the ‘All LMICs’ group, which includes all study samples except Russia and the United States. Estimates were calculated with the same procedure as above, varying only the quantity of interest; that is, one model is run for each reason why a respondent would (or would not) take the vaccine and each trusted source.

All statistical analyses, tables and figures were processed with the software R, version 4.0.4.

### Reporting Summary

Further information on research design is available in the Nature Research Reporting Summary linked to this article.

## Online content

Any methods, additional references, Nature Research reporting summaries, source data, extended data, supplementary information, acknowledgements, peer review information; details of author contributions and competing interests; and statements of data and code availability are available at 10.1038/s41591-021-01454-y.

## Supplementary information

Supplementary Information Supplementary Tables 1–13.

Reporting Summary

## Data Availability

Individual participant data (de-identified) that underlie the results reported in this Article, analytic code and replication files are available (no end date) to anyone who wishes to access the data and use it for any purpose at https://github.com/wzb-ipi/covid_vaccines_nmed. A replication exercise is available at https://wzb-ipi.github.io/covid_vaccines_nmed/.

## Code availability

All code has been deposited into the publicly available GitHub repository at https://github.com/wzb-ipi/covid_vaccines_nmed. The code and output for all analyses can be easily inspected at https://wzb-ipi.github.io/covid_vaccines_nmed/.
