# Supplementary material for: COVID-19 vaccine acceptance and hesitancy in low- and middle-income countries
Source: Nat Med. 2021 Jul 16;27(8):1385–94. doi: 10.1038/s41591-021-01454-y (PMC8363502; doi:10.1038/s41591-021-01454-y)
Supplement: Supplementary file 1 — Supplementary Tables 1–13. [file 41591_2021_1454_MOESM1_ESM.pdf]

nature portfolio

https://doi.org/10.1038/s41591-021-01454-y

Supplementary information

COVID-19 vaccine acceptance and hesitancy in low- and middle-income countries

In the format provided by the authors and unedited

Supplementary Information for *COVID-19 Vaccine Acceptance and Hesitancy in Low and Middle Income Countries*

Table S1: If a COVID-19 vaccine becomes available in [country], would you take it? Disaggregated by subgroups

		Gender		Education		Age		
**Country**	**Average acceptability**	**Female**	**Male**	**> Secondary**	**Up to Secondary**	**<25**	**25-54**	**55+**
Burkina Faso	66.5	62.1	68.4	60.8	70.1	76.0	63.2	.
	(63.5, 69.5)	(56.3, 67.9)	(65.0, 71.9)	(55.9, 65.8)	(66.4, 73.8)	(58.0, 94.0)	(53.0, 73.4)	.
Colombia	74.9	73.5	77.3	78.1	73.4	75.4	74.2	73.8
	(72.2, 77.6)	(70.1, 77.0)	(73.0, 81.7)	(73.6, 82.5)	(70.1, 76.8)	(67.5, 83.4)	(70.2, 78.3)	(65.0, 82.6)
India	84.3	82.4	84.7	87.8	85.9	77.6	85.4	83.1
	(82.3, 86.3)	(79.0, 85.8)	(82.5, 87.0)	(81.1, 94.6)	(82.5, 89.2)	(71.6, 83.6)	(83.4, 87.3)	(77.6, 88.5)
Mozambique	89.1	86.2	91.3	86.1	89.7	.	88.3	91.7
	(86.5, 91.7)	(82.5, 90.0)	(88.4, 94.1)	(81.8, 90.4)	(86.5, 92.8)	.	(85.3, 91.2)	(88.1, 95.4)
Nepal	96.6	96.4	96.4	.	.	97.8	96.6	93.8
	(95.5, 97.6)	(94.6, 98.2)	(95.1, 97.7)	.	.	(95.7, 99.8)	(95.2, 97.9)	(90.4, 97.2)
Nigeria	76.2	74.9	77.0	.	.	69.0	77.6	74.7
	(74.3, 78.2)	(71.7, 78.1)	(74.6, 79.4)	.	.	(63.3, 74.7)	(75.5, 79.7)	(65.8, 83.6)
Pakistan 1	76.1	72.2	80.1	83.6	74.0	86.3	75.6	80.8
	(70.0, 82.3)	(65.6, 78.8)	(73.8, 86.4)	(76.6, 90.5)	(67.4, 80.5)	(78.4, 94.1)	(69.3, 81.8)	(64.9, 96.7)
Pakistan 2	66.5	.	.	71.4	64.2	.	.	.
	(64.1, 68.9)	.	.	(67.3, 75.5)	(61.2, 67.1)	.	.	.
Rwanda	84.9	79.4	88.0	71.4	87.7	88.1	83.8	73.3
	(82.9, 86.8)	(75.8, 83.0)	(85.8, 90.2)	(65.5, 77.2)	(85.8, 89.7)	(85.0, 91.1)	(81.3, 86.3)	(59.2, 87.3)
Sierra Leone 1	78.0	74.1	80.1	74.4	80.2	78.0	78.3	74.0
	(75.5, 80.5)	(69.5, 78.7)	(77.2, 83.1)	(70.1, 78.7)	(77.0, 83.3)	(72.4, 83.6)	(75.4, 81.1)	(61.4, 86.6)
Sierra Leone 2	87.9	88.6	87.7	88.8	87.8	82.9	87.6	90.0
	(86.2, 89.6)	(85.7, 91.5)	(85.9, 89.5)	(85.0, 92.5)	(86.0, 89.6)	(73.9, 91.9)	(85.6, 89.5)	(87.4, 92.6)
Uganda 1	85.8	85.8	.	80.1	84.8	85.5	85.9	.
	(84.4, 87.2)	(84.4, 87.2)	.	(74.4, 85.9)	(83.2, 86.5)	(82.7, 88.4)	(84.3, 87.4)	.
Uganda 2	76.5	74.9	78.0	68.6	79.8	76.5	77.0	73.7
	(74.3, 78.7)	(71.5, 78.3)	(75.2, 80.9)	(64.3, 72.9)	(77.3, 82.2)	(71.0, 82.1)	(74.4, 79.6)	(67.3, 80.0)
All LMICs	80.3	79.2	82.6	77.4	79.8	82.8	81.1	79.1
	(74.9, 85.6)	(73.4, 85.0)	(77.4, 87.9)	(71.4, 83.4)	(74.1, 85.4)	(76.9, 88.7)	(75.6, 86.6)	(72.5, 85.7)
All LMICs (National)	78.4	75.5	80.3	74.7	79.8	80.1	77.4	74.4
	(67.9, 89.0)	(63.6, 87.5)	(70.2, 90.4)	(62.1, 87.3)	(69.8, 89.9)	(73.4, 86.7)	(65.7, 89.2)	(61.7, 87.2)
Russia	30.4	22.6	38.5	31.0	29.6	33.5	27.6	40.0
	(29.1, 31.7)	(20.9, 24.2)	(36.5, 40.5)	(29.6, 32.5)	(27.3, 32.0)	(29.2, 37.7)	(26.2, 28.9)	(35.9, 44.0)
USA	64.6	56.1	73.4	72.3	51.5	51.0	64.9	69.4
	(61.8, 67.3)	(52.1, 60.1)	(69.8, 76.9)	(69.5, 75.0)	(46.0, 57.0)	(43.5, 58.6)	(61.1, 68.7)	(64.8, 73.9)

Table S1 shows percentage of respondents willing to take the COVID-19 vaccine as plotted in Figure 1. A 95% confidence interval is shown between parentheses.

Table S2: Reasons to take the vaccine

Study	N	Protection: Self	Protection: Family	Protection: Community
Burkina Faso	651	76	42	7
		(73, 79)	(38, 46)	(5, 9)
Colombia	756	91	23	12
		(88, 93)	(20, 26)	(10, 14)
Mozambique	768	83	32	4
		(80, 86)	(27, 38)	(2, 5)
Nepal	1341	96	34	20
		(95, 98)	(32, 37)	(17, 22)
Nigeria	1424	89	35	21
		(88, 91)	(33, 38)	(19, 23)
Rwanda	1152	98	26	11
		(97, 99)	(23, 28)	(9, 13)
Sierra Leone 1	836	94	37	21
		(92, 96)	(34, 40)	(18, 23)
Sierra Leone 2	1855	91	62	21
		(88, 93)	(57, 66)	(16, 27)
Uganda 1	2885	96	36	9
		(95, 97)	(34, 38)	(8, 10)
Uganda 2	1045	96	28	11
		(95, 97)	(25, 31)	(9, 12)
All LMICs	.	91	36	14
		(86, 96)	(28, 43)	(9, 18)
Russia	5887	76	69	41
		(74, 78)	(67, 71)	(38, 43)
USA	1313	94	92	89
		(92, 95)	(90, 94)	(87, 91)

Table S2 shows percentage of respondents mentioning reasons why they would take the Covid-19 vaccine. The number of observations and percentage corresponds only to people who would take the vaccine. Respondents in all countries could give more than one reason. A 95% confidence interval is shown between parentheses. Studies India, Pakistan 1 and Pakistan 2 are not included because they either did not include the question or were not properly harmonized with the other studies.

Table S3: Reasons to take the vaccine- all categories

Study	N	Protection			If recommended by		Other
		Self	Family	Community	Health workers	Government	
Burkina Faso	651	76	42	7	6	19	2
		(73, 79)	(38, 46)	(5, 9)	(4, 8)	(16, 22)	(1, 3)
Colombia	756	91	23	12	1	2	6
		(88, 93)	(20, 26)	(10, 14)	(0, 2)	(1, 3)	(4, 7)
Mozambique	768	83	32	4	.	7	3
		(80, 86)	(27, 38)	(2, 5)	.	(5, 8)	(2, 4)
Nepal	1341	96	34	20	2	3	7
		(95, 98)	(32, 37)	(17, 22)	(1, 2)	(2, 4)	(5, 9)
Nigeria	1424	89	35	21	.	6	4
		(88, 91)	(33, 38)	(19, 23)	.	(4, 7)	(3, 5)
Rwanda	1152	98	26	11	1	5	1
		(97, 99)	(23, 28)	(9, 13)	(0, 1)	(4, 6)	(1, 2)
Sierra Leone 1	836	94	37	21	12	23	7
		(92, 96)	(34, 40)	(18, 23)	(10, 14)	(20, 25)	(5, 9)
Sierra Leone 2	1855	91	62	21	59	.	16
		(88, 93)	(57, 66)	(16, 27)	(54, 63)	.	(11, 21)
Uganda 1	2885	96	36	9	.	10	6
		(95, 97)	(34, 38)	(8, 10)	.	(9, 12)	(5, 7)
Uganda 2	1045	96	28	11	1	15	2
		(95, 97)	(25, 31)	(9, 12)	(1, 2)	(13, 17)	(1, 3)
All LMICs	.	91	36	14	12	10	5
		(86, 96)	(28, 43)	(9, 18)	(-8, 31)	(4, 16)	(2, 8)
Russia	5887	76	69	41	11	6	18
		(74, 78)	(67, 71)	(38, 43)	(10, 13)	(5, 7)	(16, 20)
USA	1313	94	92	89	.	67	.
		(92, 95)	(90, 94)	(87, 91)	.	(64, 70)	.

Table S3 shows percentage of respondents mentioning reasons why they would take the Covid-19 vaccine. The number of observations and percentage correponds only to people who would take the vaccine. Respondents in all countries could give more than one reason. A 95% confidence interval is shown between parentheses.

Table S4: Reasons to take the vaccine- by age groups

	Self			Family			Community		
**Study**	**<25**	**25-54**	**55+**	**<25**	**25-54**	**55+**	**<25**	**25-54**	**55+**
Burkina Faso	77	59	100	26	64	66	11	2	0
Conf. interval	(56, 99)	(46, 72)	(100, 100)	(4, 48)	(51, 77)	(-80, 211)	(-5, 26)	(-2, 5)	(0, 0)
n	19	57	3	19	57	3	19	57	3
Colombia	91	91	90	26	26	16	12	13	14
Conf. interval	(86, 97)	(88, 94)	(83, 97)	(17, 35)	(21, 31)	(8, 25)	(4, 20)	(9, 16)	(6, 22)
n	90	349	73	90	349	73	90	349	73
Mozambique	62	84	80	50	32	34	12	4	2
Conf. interval	(19, 106)	(81, 87)	(75, 86)	(5, 95)	(26, 38)	(27, 41)	(-17, 42)	(2, 6)	(0, 4)
n	8	571	188	8	571	188	8	571	188
Nepal	97	97	92	31	36	27	15	20	19
Conf. interval	(94, 100)	(96, 98)	(87, 97)	(25, 37)	(33, 39)	(19, 36)	(10, 20)	(17, 23)	(13, 25)
n	225	890	162	225	890	162	225	890	162
Nigeria	91	89	94	31	36	31	22	21	21
Conf. interval	(87, 95)	(87, 91)	(89, 100)	(25, 38)	(33, 39)	(20, 42)	(16, 29)	(18, 23)	(11, 31)
n	178	1175	71	178	1175	71	178	1175	71
Rwanda	98	98	100	22	28	29	10	11	10
Conf. interval	(97, 100)	(97, 99)	(100, 100)	(17, 26)	(24, 31)	(12, 46)	(7, 13)	(9, 14)	(-1, 21)
n	389	732	31	389	732	31	389	732	31
Sierra Leone 1	96	94	94	36	38	27	24	20	22
Conf. interval	(93, 99)	(92, 95)	(86, 102)	(29, 44)	(34, 41)	(12, 42)	(17, 31)	(16, 23)	(8, 36)
n	167	632	37	167	632	37	167	632	37
Sierra Leone 2	87	90	93	52	62	62	29	22	18
Conf. interval	(78, 97)	(88, 92)	(89, 97)	(39, 66)	(58, 67)	(56, 67)	(16, 42)	(16, 28)	(12, 25)
n	63	1376	396	63	1376	396	63	1376	396
Uganda 1	96	96	.	34	36	.	9	9	.
Conf. interval	(95, 98)	(96, 97)	.	(30, 39)	(34, 39)	.	(6, 11)	(8, 11)	.
n	526	2218	.	526	2218	.	526	2218	.
Uganda 2	97	96	97	20	30	28	8	11	13
Conf. interval	(94, 99)	(95, 97)	(94, 100)	(14, 26)	(27, 33)	(21, 36)	(4, 11)	(9, 13)	(7, 19)
n	173	749	123	173	749	123	173	749	123
All LMICs	89	89	93	33	39	36	15	13	13
Conf. interval	(81, 97)	(81, 98)	(89, 98)	(25, 41)	(29, 48)	(23, 48)	(10, 20)	(8, 18)	(7, 19)
n	1838	8749	1084	1838	8749	1084	1838	8749	1084
Russia	67	76	81	74	68	68	46	40	38
Conf. interval	(59, 74)	(73, 78)	(76, 87)	(68, 81)	(66, 71)	(62, 74)	(38, 54)	(38, 43)	(32, 44)
n	552	5108	227	552	5108	227	552	5108	227
USA	92	91	97	89	91	94	90	89	89
Conf. interval	(88, 96)	(89, 94)	(95, 99)	(83, 95)	(88, 93)	(91, 97)	(85, 95)	(86, 92)	(85, 93)
n	153	687	473	153	687	473	153	687	473

Table S4 shows percentage of respondents mentioning reasons why they would take the Covid-19 vaccine by age groups. The number of observations and percentage correponds only to people who would take the vaccine. Respondents in all countries could give more than one reason. A 95% confidence interval is shown between parentheses.

Table S5: Reasons not to take the vaccine

Study	N	Concerned about side effects	Concerned about getting coronavirus from the vaccine	Not concerned about getting seriously ill	Doesn't think vaccines are effective	Doesn't think Coronavirus outbreak is as serious as people say	Doesn't like needles	Allergic to vaccines	Won't have time to get vaccinated	Mentions a conspiracy theory	Other reasons
Burkina Faso	325	40.9	8.0	7.4	19.5	13.5	3.5	1.5	0.9	17.9	8.7
		(35.5, 46.3)	(5.0, 11.0)	(4.5, 10.2)	(15.1, 23.8)	(9.8, 17.2)	(1.5, 5.6)	(0.2, 2.8)	(-0.1, 1.9)	(13.7, 22.1)	(5.6, 11.8)
Colombia	202	31.0	18.1	8.0	10.2	2.3	0.6	0.4	0.5	10.0	31.6
		(24.4, 37.6)	(12.7, 23.4)	(3.9, 12.0)	(5.9, 14.5)	(0.3, 4.3)	(-0.6, 1.8)	(-0.4, 1.3)	(-0.5, 1.5)	(5.8, 14.2)	(25.1, 38.2)
Mozambique	74	.	.	2.7	29.7	.	.	.	.	.	21.6
		.	.	(-0.7, 6.1)	(18.6, 40.8)	.	.	.	.	.	(12.2, 31.0)
Nepal	48	9.3	7.9	20.4	15.2	15.7	4.4	1.8	.	2.8	12.1
		(0.3, 18.2)	(-0.4, 16.3)	(6.7, 34.1)	(3.2, 27.2)	(4.0, 27.3)	(-1.9, 10.6)	(-1.9, 5.5)	.	(-1.5, 7.2)	(0.8, 23.5)
Nigeria	410	21.5	26.1	15.9	9.3	.	.	0.2	.	4.9	26.8
		(17.5, 25.5)	(21.8, 30.4)	(12.3, 19.4)	(6.4, 12.1)	.	.	(-0.2, 0.7)	.	(2.8, 7.0)	(22.5, 31.1)
Pakistan 1	441	23.0	21.9	29.4	26.0	22.1	11.5	.	.	13.2	19.6
		(15.1, 30.8)	(14.3, 29.4)	(20.9, 37.9)	(18.0, 34.0)	(12.8, 31.3)	(5.5, 17.4)	.	.	(7.1, 19.4)	(10.4, 28.8)
Rwanda	70	38.6	10.1	18.7	21.5	5.8	7.0	5.6	.	21.3	25.8
		(26.9, 50.3)	(2.8, 17.3)	(9.3, 28.1)	(11.6, 31.4)	(0.1, 11.4)	(0.9, 13.2)	(0.1, 11.1)	.	(11.5, 31.1)	(15.3, 36.3)
Sierra Leone 1	234	53.5	37.9	14.6	7.5	4.2	3.0	0.9	4.0	20.3	5.7
		(47.1, 59.9)	(31.6, 44.2)	(10.1, 19.2)	(4.2, 10.9)	(1.6, 6.8)	(0.8, 5.2)	(-0.4, 2.2)	(1.4, 6.5)	(15.1, 25.5)	(2.8, 8.7)
Sierra Leone 2	254	57.9	.	.	17.3	.	5.1	.	0.0	3.5	24.8
		(50.1, 65.7)	.	.	(11.9, 22.7)	.	(2.5, 7.8)	.	(0.0, 0.0)	(1.3, 5.7)	(19.3, 30.3)
Uganda 1	289	85.1	.	3.8	24.2	1.7	1.7	.	1.0	.	8.0
		(80.7, 89.6)	.	(1.7, 5.9)	(19.2, 29.2)	(0.2, 3.2)	(0.2, 3.2)	.	(-0.1, 2.2)	.	(4.9, 11.0)
Uganda 2	319	47.3	10.7	5.0	31.0	4.1	1.6	0.3	.	10.3	6.0
		(42.2, 52.5)	(7.1, 14.2)	(2.7, 7.3)	(25.9, 36.2)	(1.9, 6.2)	(0.2, 2.9)	(-0.3, 0.9)	.	(7.0, 13.7)	(3.4, 8.5)
All LMICs	.	40.8	17.6	12.6	19.2	8.7	4.3	1.5	1.3	11.6	17.3
		(25.3, 56.3)	(8.7, 26.5)	(6.4, 18.8)	(13.8, 24.7)	(2.4, 14.9)	(1.7, 6.8)	(-0.2, 3.3)	(-0.6, 3.2)	(6.1, 17.0)	(11.0, 23.7)
Russia	16238	36.8	13.9	5.4	29.6	6.4	3.7	10.2	1.0	21.4	5.1
		(35.2, 38.4)	(12.8, 15.1)	(4.6, 6.1)	(28.1, 31.1)	(5.6, 7.3)	(3.1, 4.3)	(9.2, 11.2)	(0.7, 1.4)	(20.1, 22.8)	(4.4, 5.8)
USA	462	79.3	.	39.3	46.8	.	.	.	.	6.0	49.1
		(74.6, 84.0)	.	(33.5, 45.0)	(41.0, 52.6)	.	.	.	.	(3.4, 8.7)	(43.3, 54.9)

Table S5 shows percentage of respondents mentioning reasons why they would not take the Covid-19 vaccine. The number of observations and percentage correponds only to people who would NOT take the vaccine. Respondents in all countries could give more than one reason. A 95% confidence interval is shown between parentheses.

Table S6: COVID-19 Vaccination Decision-making: most trusted source

Study	N	Take vaccine?	Health workers	Government or Ministry of Health	Family or friends	Famous person, religious leader or traditional healers	Newspapers, radio or online groups	Other	Don't know or Refuse
Burkina Faso	651	Yes	57.1	15.1	19.6	0.9	2.0	4.8	0.4
			(53.3, 60.9)	(12.4, 17.9)	(16.5, 22.7)	(0.2, 1.6)	(0.9, 3.1)	(3.2, 6.4)	(-0.1, 0.9)
Burkina Faso	325	No	40.7	8.5	16.2	3.7	1.6	25.1	4.2
			(35.3, 46.1)	(5.5, 11.6)	(12.1, 20.2)	(1.6, 5.7)	(0.2, 3.0)	(20.3, 29.8)	(2.0, 6.4)
Burkina Faso	976	All	51.6	12.9	18.4	1.8	1.9	11.6	1.7
			(48.5, 54.8)	(10.8, 15.0)	(16.0, 20.9)	(1.0, 2.7)	(1.0, 2.7)	(9.6, 13.6)	(0.9, 2.5)
Colombia	756	Yes	41.4	12.7	36.9	0.9	1.7	.	6.3
			(37.8, 45.0)	(10.3, 15.2)	(33.4, 40.4)	(0.2, 1.5)	(0.8, 2.7)	.	(4.6, 8.1)
Colombia	202	No	31.5	7.6	35.5	5.3	1.4	.	18.8
			(24.9, 38.1)	(3.8, 11.3)	(28.8, 42.1)	(2.2, 8.4)	(-0.2, 3.0)	.	(13.2, 24.3)
Colombia	958	All	39.3	11.6	36.6	1.8	1.7	.	8.9
			(36.2, 42.5)	(9.6, 13.7)	(33.5, 39.7)	(1.0, 2.6)	(0.9, 2.5)	.	(7.1, 10.7)
Nepal	1341	Yes	44.7	0.7	36.2	16.1	0.4	0.5	1.3
			(40.9, 48.6)	(0.3, 1.1)	(33.5, 39.0)	(13.1, 19.1)	(0.0, 0.9)	(0.1, 0.8)	(0.7, 2.0)
Nepal	48	No	30.2	2.1	18.7	16.8	0.0	1.0	31.2
			(14.6, 45.9)	(-2.1, 6.2)	(5.6, 31.7)	(4.0, 29.6)	(0.0, 0.0)	(-1.1, 3.2)	(13.6, 48.9)
Nepal	1389	All	44.2	0.8	35.6	16.1	0.4	0.5	2.4
			(40.5, 47.9)	(0.3, 1.2)	(32.9, 38.3)	(13.3, 18.9)	(0.0, 0.8)	(0.1, 0.8)	(1.5, 3.3)
Nigeria	1424	Yes	63.8	21.6	6.3	5.1	.	2.6	0.6
			(61.3, 66.3)	(19.4, 23.7)	(5.0, 7.5)	(4.0, 6.3)	.	(1.8, 3.4)	(0.2, 1.0)
Nigeria	410	No	37.6	5.6	13.9	17.8	.	8.5	16.6
			(32.9, 42.3)	(3.4, 7.8)	(10.5, 17.3)	(14.1, 21.5)	.	(5.8, 11.3)	(13.0, 20.2)
Nigeria	1834	All	58.0	18.0	8.0	8.0	.	3.9	4.2
			(55.7, 60.2)	(16.2, 19.8)	(6.7, 9.2)	(6.7, 9.2)	.	(3.0, 4.8)	(3.3, 5.1)
Rwanda	1152	Yes	23.8	27.4	15.1	1.0	0.7	32.0	0.1
			(21.3, 26.2)	(24.9, 30.0)	(13.0, 17.2)	(0.4, 1.5)	(0.2, 1.2)	(29.3, 34.7)	(-0.1, 0.2)
Rwanda	70	No	10.1	15.6	12.8	2.9	0.0	53.2	5.5
			(2.8, 17.4)	(6.9, 24.3)	(4.8, 20.8)	(-1.1, 6.9)	(0.0, 0.0)	(41.2, 65.1)	(0.1, 11.0)
Rwanda	1222	All	23.0	26.7	15.0	1.1	0.6	33.2	0.4
			(20.6, 25.3)	(24.3, 29.2)	(13.0, 17.0)	(0.5, 1.7)	(0.2, 1.1)	(30.5, 35.8)	(0.0, 0.8)

Table S6: COVID-19 Vaccination Decision-making: most trusted source *(continued)*

Study	N	Take vaccine?	Health workers	Government or Ministry of Health	Family or friends	Famous person, religious leader or traditional healers	Newspapers, radio or online groups	Other	Don't know or Refuse
Sierra Leone 1	836	Yes	47.6	36.9	7.3	3.8	0.5	3.1	0.8
			(44.2, 51.0)	(33.6, 40.2)	(5.5, 9.1)	(2.5, 5.1)	(0.0, 1.0)	(1.9, 4.2)	(0.2, 1.4)
Sierra Leone 1	234	No	31.1	17.1	12.1	7.7	0.5	29.4	2.2
			(25.1, 37.1)	(12.2, 21.9)	(7.9, 16.3)	(4.3, 11.2)	(-0.4, 1.3)	(23.5, 35.3)	(0.3, 4.1)
Sierra Leone 1	1070	All	44.0	32.5	8.4	4.7	0.5	8.9	1.1
			(41.0, 46.9)	(29.7, 35.4)	(6.7, 10.0)	(3.4, 6.0)	(0.1, 0.9)	(7.1, 10.6)	(0.5, 1.8)
Sierra Leone 2	1855	Yes	94.1	.	3.0	0.9	0.1	1.9	0.0
			(92.5, 95.7)	.	(2.0, 4.0)	(0.3, 1.5)	(-0.1, 0.2)	(1.2, 2.7)	(0.0, 0.0)
Sierra Leone 2	254	No	54.7	.	3.9	7.5	0.0	33.9	0.0
			(46.5, 62.9)	.	(1.4, 6.5)	(2.9, 12.0)	(0.0, 0.0)	(26.3, 41.4)	(0.0, 0.0)
Sierra Leone 2	2109	All	89.3	.	3.1	1.7	0.0	5.8	0.0
			(87.2, 91.5)	.	(2.2, 4.1)	(0.8, 2.6)	(0.0, 0.1)	(4.4, 7.2)	(0.0, 0.0)
Uganda 2	1045	Yes	38.3	36.5	9.8	7.0	5.0	3.5	0.0
			(35.5, 41.1)	(33.5, 39.4)	(7.9, 11.6)	(5.4, 8.6)	(3.6, 6.3)	(2.5, 4.6)	(0.0, 0.0)
Uganda 2	319	No	24.5	19.1	8.5	7.8	7.5	32.0	0.6
			(19.9, 29.0)	(14.5, 23.7)	(5.4, 11.5)	(4.8, 10.9)	(4.5, 10.5)	(26.7, 37.3)	(-0.2, 1.5)
Uganda 2	1364	All	35.0	32.4	9.5	7.2	5.6	10.2	0.1
			(32.7, 37.4)	(29.9, 35.0)	(7.9, 11.1)	(5.8, 8.6)	(4.3, 6.8)	(8.6, 11.8)	(-0.1, 0.3)
All LMICs	.	Yes	51.3	21.6	16.8	4.5	1.5	6.9	1.2
			(33.7, 68.9)	(9.4, 33.8)	(5.7, 27.9)	(0.1, 8.8)	(-0.1, 3.1)	(-3.4, 17.2)	(-0.6, 3.0)
All LMICs	.	No	32.5	10.8	15.2	8.7	1.6	26.1	9.9
			(21.8, 43.3)	(4.8, 16.8)	(7.4, 23.0)	(4.0, 13.4)	(-0.9, 4.1)	(10.2, 42.1)	(0.6, 19.2)
All LMICs	.	All	48.1	19.3	16.8	5.3	1.5	10.6	2.4
			(31.6, 64.5)	(8.3, 30.3)	(6.1, 27.5)	(1.0, 9.6)	(-0.2, 3.3)	(0.7, 20.5)	(-0.1, 4.9)
Russia	5887	Yes	47.1	24.4	16.5	2.0	4.1	5.8	.
			(44.6, 49.7)	(22.2, 26.7)	(14.6, 18.5)	(1.2, 2.8)	(3.1, 5.1)	(4.5, 7.0)	.
Russia	16238	No	31.1	6.9	33.1	2.2	5.3	21.3	.
			(29.6, 32.7)	(6.1, 7.8)	(31.5, 34.7)	(1.7, 2.8)	(4.5, 6.0)	(20.0, 22.7)	.
Russia	22125	All	36.0	12.3	28.1	2.2	4.9	16.6	.
			(34.7, 37.3)	(11.3, 13.2)	(26.8, 29.3)	(1.7, 2.6)	(4.3, 5.5)	(15.6, 17.6)	.

Table S6: COVID-19 Vaccination Decision-making: most trusted source *(continued)*

Study	N	Take vaccine?	Health workers	Government or Ministry of Health	Family or friends	Famous person, religious leader or traditional healers	Newspapers, radio or online groups	Other	Don't know or Refuse
USA	1313	Yes	38.1	33.0	8.7	1.7	.	18.6	0.0
			(34.8, 41.5)	(29.8, 36.1)	(6.7, 10.7)	(0.7, 2.6)	.	(16.1, 21.1)	(0.0, 0.0)
USA	462	No	25.3	21.3	18.7	4.2	.	30.3	0.2
			(20.4, 30.3)	(16.6, 26.0)	(13.9, 23.4)	(1.6, 6.9)	.	(25.0, 35.6)	(-0.2, 0.7)
USA	1775	All	34.5	29.7	11.5	2.4	.	21.9	0.1
			(31.7, 37.3)	(27.0, 32.3)	(9.5, 13.4)	(1.4, 3.4)	.	(19.5, 24.2)	(-0.1, 0.2)

Table S6 shows percentage of respondents that mention actors who they would trust the most to help them decide whether to get a COVID-19 vaccine. For all countries the questions was asked regardless if respondent would take a vaccine, would not take it, does not know or does not respond. For India respondents were able to mention more than one actor, for the rest of countries only one actor was allowed. While rows should sum to 100%, rounding makes number slightly above or below. A 95% confidence interval is shown between parentheses.

Table S7: Differences in means

Estimate	Std.error	P-value	Degrees of freedom	Baseline category	Variable
0.04	0.01	0.00	10	Male	Gender (Female)
-0.02	0.02	0.43	10	<25	Age (25-54)
-0.02	0.02	0.36	10	<25	Age (55+)
0.02	0.03	0.38	10	Up to secondary	Education (Secondary +)

Table S7 shows the results of subgroup mean differences. Subgroup differences were generated considering only LMICs. p-values come from a two-sided t-test from a linear regression.

Table S8: Observations and missingness patterns

Country	N obs	Take vaccine	Gender	Education	Age
Burkina Faso	977	99.90	100.00	100.00	12.28
Colombia	1,012	94.66	100.00	99.90	68.18
India	1,680	100.00	100.00	20.24	100.00
Mozambique	862	97.68	100.00	96.06	99.77
Nepal	1,389	100.00	95.32	0.00	95.32
Nigeria	1,868	98.18	100.00	0.00	100.00
Pakistan 1	1,633	98.96	99.76	99.27	100.00
Pakistan 2	1,492	99.87	0.00	100.00	0.00
Russia	22,125	100.00	100.00	100.00	100.00
Rwanda	1,355	90.18	100.00	100.00	100.00
Sierra Leone 1	1,070	100.00	100.00	97.01	100.00
Sierra Leone 2	2,110	99.95	100.00	100.00	98.91
Uganda 1	3,362	94.41	100.00	81.47	95.12
Uganda 2	1,366	99.85	100.00	100.00	100.00
USA	1,959	90.61	100.00	100.00	100.00

Table S8 show the proportion of observations that are not missing values for each variable included in Figure 1.

Table S9: Differences between groups within studies

Country	Variable	Baseline category	Group	Estimate	Std. Error	P-value	Degrees of freedom	N Obs
Burkina Faso	Age	<25	25-54	-0.13	0.10	0.21	119	120
Colombia	Age	<25	25-54	-0.01	0.04	0.79	689	690
India	Age	<25	25-54	0.08	0.03	0.01	141	1,680
Mozambique	Age	<25	25-54	-0.12	0.01	0.00	162	860
Nepal	Age	<25	25-54	-0.01	0.01	0.32	89	1,324
Nigeria	Age	<25	25-54	0.09	0.03	0.01	1,867	1,868
Pakistan 1	Age	<25	25-54	-0.11	0.04	0.00	105	1,633
Russia	Age	<25	25-54	-0.06	0.02	0.01	22,124	22,125
Rwanda	Age	<25	25-54	-0.04	0.02	0.03	1,354	1,355
Sierra Leone 1	Age	<25	25-54	0.00	0.03	0.94	1,069	1,070
Sierra Leone 2	Age	<25	25-54	0.05	0.04	0.30	190	2,087
Uganda 1	Age	<25	25-54	0.00	0.02	0.83	497	3,198
Uganda 2	Age	<25	25-54	0.00	0.03	0.89	309	1,366
USA	Age	<25	25-54	0.14	0.04	0.00	1,958	1,959
Burkina Faso	Age	<25	55+	-0.15	0.24	0.53	119	120
Colombia	Age	<25	55+	-0.02	0.06	0.79	689	690
India	Age	<25	55+	0.05	0.04	0.19	141	1,680
Mozambique	Age	<25	55+	-0.08	0.02	0.00	162	860
Nepal	Age	<25	55+	-0.04	0.02	0.06	89	1,324
Nigeria	Age	<25	55+	0.06	0.05	0.28	1,867	1,868
Pakistan 1	Age	<25	55+	-0.06	0.07	0.45	105	1,633
Russia	Age	<25	55+	0.07	0.03	0.03	22,124	22,125
Rwanda	Age	<25	55+	-0.15	0.07	0.04	1,354	1,355
Sierra Leone 1	Age	<25	55+	-0.04	0.07	0.56	1,069	1,070
Sierra Leone 2	Age	<25	55+	0.07	0.05	0.12	190	2,087
Uganda 2	Age	<25	55+	-0.03	0.04	0.47	309	1,366
USA	Age	<25	55+	0.18	0.04	0.00	1,958	1,959
Burkina Faso	Education	Secondary +	Up to secondary	0.09	0.03	0.00	976	977
Colombia	Education	Secondary +	Up to secondary	-0.05	0.03	0.10	1,010	1,011
India	Education	Secondary +	Up to secondary	-0.02	0.04	0.59	100	340
Mozambique	Education	Secondary +	Up to secondary	0.04	0.03	0.17	160	828
Pakistan 1	Education	Secondary +	Up to secondary	-0.10	0.04	0.01	105	1,621
Pakistan 2	Education	Secondary +	Up to secondary	-0.07	0.03	0.00	1,491	1,492
Russia	Education	Secondary +	Up to secondary	-0.01	0.01	0.31	22,124	22,125
Rwanda	Education	Secondary +	Up to secondary	0.16	0.03	0.00	1,354	1,355
Sierra Leone 1	Education	Secondary +	Up to secondary	0.06	0.03	0.03	1,037	1,038
Sierra Leone 2	Education	Secondary +	Up to secondary	-0.01	0.02	0.63	190	2,110
Uganda 1	Education	Secondary +	Up to secondary	0.05	0.03	0.12	494	2,739
Uganda 2	Education	Secondary +	Up to secondary	0.11	0.03	0.00	309	1,366
USA	Education	Secondary +	Up to secondary	-0.21	0.03	0.00	1,958	1,959
Burkina Faso	Gender	Female	Male	0.06	0.03	0.06	976	977
Colombia	Gender	Female	Male	0.04	0.03	0.18	1,011	1,012
India	Gender	Female	Male	0.02	0.02	0.22	141	1,680
Mozambique	Gender	Female	Male	0.05	0.02	0.02	162	862
Nepal	Gender	Female	Male	0.00	0.01	0.98	89	1,324
Nigeria	Gender	Female	Male	0.02	0.02	0.30	1,867	1,868
Pakistan 1	Gender	Female	Male	0.08	0.02	0.00	105	1,629
Russia	Gender	Female	Male	0.16	0.01	0.00	22,124	22,125
Rwanda	Gender	Female	Male	0.09	0.02	0.00	1,354	1,355
Sierra Leone 1	Gender	Female	Male	0.06	0.03	0.03	1,069	1,070
Sierra Leone 2	Gender	Female	Male	-0.01	0.02	0.56	190	2,110
Uganda 2	Gender	Female	Male	0.03	0.02	0.17	309	1,366
USA	Gender	Female	Male	0.17	0.03	0.00	1,958	1,959

Table S9 shows differences of means between groups within single studies. Estimates are calculated through OLS and represent the difference in the average acceptance rate between the subgroup in column Group and that in column Baseline category. p-values come from a two-sided t-test from a linear regression.

Table S10: Question wording and answer options: vaccine acceptance

Study	Question Fig. 1	Recoding Fig. 1
Burkina Faso	If a COVID-19 vaccine became available in Burkina Faso, would you take it?	Yes; No; Don't know; Refuse
Colombia	If a COVID-19 vaccine became available in Colombia, would you take it?	Yes; No
India	If a vaccine for coronavirus gets introduced, would you like to get it?	Yes, only for free; Yes, even if I have to pay; No
Mozambique	When a COVID-19 vaccine becomes available in the future, would you take it?	Yes; No; Refuse
Nepal	Should a vaccine against COVID become available in Nepal, would you take it?	Yes; No
Nigeria	If a COVID-19 vaccine became available in Niger, would you take it?	Yes/Agree; No/Disagree
Pakistan 1	If a vaccine against the coronavirus becomes available, do you plan to get vaccinated?	Yes; No; Don't know; Refuse
Pakistan 2	If a vaccine against the coronavirus becomes available, do you plan to get vaccinated?	Absolutely yes; Yes; Neutral; No; Absolutely no
Russia	If a COVID-19 vaccine became available in Russia, would you take it?	Yes, if a Russian vaccine will be available; Yes, if an imported vaccine will be available; No; Not sure
Rwanda	If a COVID-19 vaccine became available in Rwanda, would you take it?	Yes; No
Sierra Leone 1	If a COVID-19 vaccine became available in Sierra Leone, would you take it?	Yes; No
Sierra Leone 2	Should a vaccine against COVID become available in Sierra Leone, would you take it?	Yes; No
Uganda 1	When a COVID-19 vaccine becomes available in Uganda, would you take it?	Yes; No
Uganda 2	If a COVID-19 vaccine becomes available in Uganda, would you take it?	Yes; No; Don't know; Refuse
USA	If a COVID-19 vaccine becomes available in the United States, would you take it?	Definitely yes; Probably yes; Probably not; Definitely not, Refuse

Table S10 presents question wording and answer options from answers used in Figure 1 to get estimated vaccine acceptance. Answer options are separated by a semicolon. In India options 'Yes, only for free' and 'Yes, even if I have to pay' are both recoded as 'Yes'. In Pakistan 2, 'Absolutely yes' is recoded as 'Yes', 'Neutral' is recoded as 'Don't know' and 'Absolutely no' is recoded as 'No'. In Russia, 'Yes, if a Russian vaccine will be available' and 'Yes, if an imported vaccine will be available' are both recoded as 'Yes'. In USA 'Definitely yes' and 'Probably yes' are recoded as 'Yes', and 'Probably not' and 'Definitely not' are recoded as 'No'.

Table S11: Question wording and answer options: reasons to take vaccine

Study	Question Tab. 2	Protection: self	Protection: family	Protection: community
Burkina Faso	Why would you take it?	Protection: self (general); Protection: self, chronic condition	Protection: family	Protection: community
Colombia	Why would you take it?	Protection: self (general); Protection: self, chronic condition	Protection: family	Protection: community
Mozambique	Why would you take it?	I want to protect myself from having COVID-19 in the future	I want to protect my family/members of my household against having COVID-19 in the future	I want to protect my community against having COVID-19 in the future
Nepal	Why would you take it?	Protection: self (general); Protection: self, chronic condition/ vulnerable to covid	Protection: family	Protection: community
Nigeria	Why would you take it?	I want to protect myself from having COVID-19 in the future	I want to protect my family/members of my household against having COVID-19 in the future	I want to protect my community against having COVID-19 in the future
Russia	Why would you take it?	Protection: self	Protection: family	Protection: community
Rwanda	Why would you take it?	Protection: self (general); Protection: self, chronic condition	Protection: family	Protection: community
Sierra Leone 1	Why would you take it?	Protection: self (general); Protection: self, chronic condition	Protection: family	Protection: community
Sierra Leone 2	Why would you take it?	I will take a vaccine to protect myself from having COVID-19 in the future	I will take a vaccine to protect my family/members of my household against having COVID-19 in the future	I will take a vaccine to protect my community against having COVID-19 in the future
Uganda 1	Why would you take it?	Protect myself from having COVID-19	Protect my family/members of my household against COVID-19	Protect my community against COVID-19
Uganda 2	Why would you take it?	Protection: self (general); Protection: self, chronic condition/ vulnerable to Covid	Protection: family	Protection: community
USA	Why would you take it?	To protect myself from COVID-19 infection	To protect my family from COVID-19 infection	To protect my community from COVID-19 infection

Table S11 presents question wording and answer options used in Table S2 to get an estimated percentage of reasons to take the COVID-19 vaccine. Columns 'Protection: self', 'Protection: family' and 'Protection: community' show the answer options that were recoded in each category. Answer options are separated by a semicolon.

Table S12: Question wording and answer options: reasons not to take the vaccine

Study	Question Fig. 2	Concerned about side effects	Concerned about getting COVID-19 from the vaccine	Not concerned about getting seriously ill	Doesn't think vaccines are effective	Doesn't think COVID-19 outbreak is as serious as people say	Doesn't like needles	Allergic to vaccines	Won't have time to get vaccinated	Mentions a conspiracy theory	Other reasons
Burkina Faso	Why would you not take it?	.	Concerned about getting coronavirus from the vaccine	Not concerned about getting seriously ill	Doesn't think vaccines work very well	Coronavirus outbreak is not as serious as people say	Doesn't like needles	Allergic to vaccines	Won't have time to get vaccinated	Conspiracy theory	Other reason
Colombia	Why would you not take it?	.	Concerned about getting coronavirus from the vaccine	Not concerned about getting seriously ill	Doesn't think vaccines work very well	Coronavirus outbreak is not as serious as people say	Doesn't like needles	Allergic to vaccines	Won't have time to get vaccinated	Conspiracy theory	Other reason; Already immune; Doesn't have symptoms
Mozambique	Why would you not take it?	.	.	I am not concerned about the risk associated with me/my relatives getting COVID-19	I don't think vaccines are effective	The coronavirus outbreak is not as serious as people say it is	I don't like needles	.	I won't have time to go get vaccinated	.	Other
Nepal	Why would you not take it?	I would be concerned about the side effects from the vaccine	I would be concerned about getting infected with coronavirus from the vaccine	I'm not concerned about getting seriously ill from the virus	I don't think vaccines work very well	The coronavirus outbreak is not as serious as people say it is	I don't like needles	I'm allergic to vaccines	I won't have time to go get vaccinated	I think there is a conspiracy theory with vaccinations	Other
Nigeria	Why would you not take it?	I would be concerned about the side effects from the vaccine	I would be concerned about getting infected with coronavirus from the vaccine	I'm not concerned about getting seriously ill from the virus	I don't think vaccines work very well	The coronavirus outbreak is not as serious as people say it is	I don't like needles	I'm allergic to vaccines	I won't have time to go get vaccinated	The virus is a hoax / does not exist; The vaccine has microchips/ tracking devices	Other; Religious / community leaders advising me not to take it
Pakistan 1	Why would you not take it?	I am concerned about side effects from the vaccine	I would be concerned about getting infected with coronavirus from the vaccine	I don't consider myself or my family members at risk of getting seriously ill	I don't think the vaccine would work well	The coronavirus infection is just like the flu and doesn't warrant a vaccine	I don't like needles	.	.	Vaccines are just Western conspiracies to stunt the growth of Muslims	Muslims are prohibited from taking a vaccine before a disease is contracted
Russia	Why would you not take it?	Afraid of side effects	Afraid of getting infected with coronavirus from the vaccine	Not concerned with getting seriously ill from the virus	Don't think vaccines are effective	Coronavirus outbreak is not as serious as people say it is	Afraid of needles	Can get allergic reaction	Don't have time to get vaccinated	Hoax: Virus don't exist; Hoax: Virus was designed so vaccines won't work; Profit motivation: pharmaceutical companies; Control: contain things that control our minds; Global politics: China can take advantage	Other; I already had coronavirus and don't need a vaccine
Rwanda	Why would you not take it?	.	Concerned about getting coronavirus from the vaccine	Not concerned about getting seriously ill	Doesn't think vaccines work very well	Coronavirus outbreak is not as serious as people say	Doesn't like needles	Allergic to vaccines	Won't have time to get vaccinated	Conspiracy theory	Other reason; Already immune; Doesn't have symptoms
Sierra Leone 1	Why would you not take it?	.	Concerned about getting coronavirus from the vaccine	Not concerned about getting seriously ill	Doesn't think vaccines work very well	Coronavirus outbreak is not as serious as people say	Doesn't like needles	Allergic to vaccines	Won't have time to get vaccinated	Conspiracy theory	Other reason
Sierra Leone 2	Why would you not take it?	I will not take a vaccine because I am concerned about side effects	I will not take a vaccine because I am not concerned about the risk associated with me/my relatives getting COVID-19	I will not take a vaccine because I am not concerned about the risk associated with me/my relatives getting COVID-19	I will not take a vaccine because they are not effective	.	I will not take a vaccine because I don't like needles	.	I will not take a vaccine because I don't have time	I will not take a vaccine because I don't think COVID exists	I will not take a vaccine because of other reasons; I will not take a vaccine because my community objects it; I will not take a vaccine because I don't have symptoms; I will not take a vaccine because I am immune; I will not take a vaccine because it is provided by foreign aid; I will not take a vaccine because I don't know what a vaccine is
Uganda 1	Why would you not take it?	Concerned about the side effects from the vaccine/vaccines	I am not worried that my relatives will get COVID-19	I am not worried that my relatives will get COVID-19	I don't think vaccines are effective	Coronavirus is not as serious as people say it is	I don't like needles	.	I won't have time to go get vaccinated	.	Other; It will cost too much
Uganda 2	Why would you not take it?	I would be concerned about the side effects from the vaccine	I would be concerned about getting infected with coronavirus from the vaccine	I'm not concerned about getting seriously ill from the virus	I don't think vaccines work very well	The coronavirus outbreak is not as serious as people say it is	I don't like needles	I'm allergic to vaccines	I won't have time to go get vaccinated	I think there is a conspiracy theory with vaccinations	Other
USA	Why would you not take it?	I am concerned about possible side effects	.	I am not concerned about getting the virus	I don't think vaccines are effective	.	.	.	.	Mentions a conspiracy theory (recoded from responses in "Other" category)	Cost or difficulty of getting the vaccine

Table S12 presents question wording and answer options used in Figure 2 to get an estimated percentage of reasons not to take the COVID-19 vaccine. Columns 3-10 show the answer options that were recoded in each category. Answer options are separated by a semicolon.

Table S13: Question wording and answer options: trusted actors and institutions

Study	Question Fig. 3	Health workers	Government or Ministry of Health	Family or friends	Famous person, religious leader or traditional healers	Newspapers, radio or online groups	Other
Burkina Faso	Which of the following people would you trust MOST to help you decide whether you would get a COVID-19 vaccine, if one becomes available?	Doctors or other staff at a community health clinic	Advice from Ministry of Health	Family members; Friends you see and talk to; Friends you-'ve made online	Famous person; Religious leaders; Traditional Healers	Traditional media (newspaper, radio); Online medical discussion groups	Other/ Someone else
Colombia	Which of the following people would you trust MOST to help you decide whether you would get a COVID-19 vaccine, if one becomes available?	Doctors or other staff at a community health clinic	Advice of the Instituto Nacional de Salud	Family members; Friends you see and talk to; Friends you've made online	Famous person; Religious leaders; Traditional Healers	Traditional media (newspaper, radio); Online medical discussion groups	Other/ Someone else
Nepal	Which of the following people would you trust MOST to help you decide whether you would get a COVID-19 vaccine, if one becomes available?	Doctors or other staff at a community health clinic	Advice of the national health service	Family members; Friends you see and talk to	Famous person; Religious leaders; Traditional healers	Traditional media (newspaper, radio); Online medical discussion groups	None of these/ Someone else; Advice of the WHO
Nigeria	Which of the following people would you trust MOST to help you decide whether you would get a COVID-19 vaccine, if one becomes available?	Medical professionals like doctors	NCDC; Government officials	Family members and friends	Religious leaders	.	Some other sourcer; Other community leaders
Russia	Which of the following people would you trust MOST to help you decide whether you would get a COVID-19 vaccine, if one becomes available?	Health workers	Government; Health Ministry	Family; Friends	Famous people; Religious leaders	Traditional media; Online medical discussion groups	Other
Rwanda	Which of the following people would you trust MOST to help you decide whether you would get a COVID-19 vaccine, if one becomes available?	Doctors or other staff at a community health clinic	Advice of the Ministry of Health	Family members; Friends you see and talk to; Friends you've made online	Famous person; Religious leaders; Traditional healers	Traditional media (newspaper, radio); Online medical discussion groups	None of these/ Someone else; Myself
Sierra Leone 1	Which of the following people would you trust MOST to help you decide whether you would get a COVID-19 vaccine, if one becomes available?	Doctors or other staff at a community health clinic	Advice of the Ministry of Health and Sanitation	Family members; Friends you see and talk to; Friends you've made online	Famous person; Religious leaders; Traditional healers	Traditional media (newspaper, radio); Online medical discussion groups	None of these/ Someone else; I do trust NOBODY
Sierra Leone 2	Which of the following people would you trust MOST to help you decide whether you would get a COVID-19 vaccine, if one becomes available?	A doctor, nurse or other staff at a community health clinic; A country medical staff	.	Family; Friends you see and talk to; Friends you've made online	A famous person; A religious leader; A traditional healer	Online medical discussion groups	None of these/ Someone else
Uganda 2	Which of the following people would you trust MOST to help you decide whether you would get a COVID-19 vaccine, if one becomes available?	Doctors or other staff at a community health clinic	Advice of the national health service	Family; Friends you see and talk to; Friends you've made online	Famous person; Religious leaders; Traditional healers	Traditional media (newspaper, radio); Online medical discussion groups	None of these; Someone else
USA	Which of the following people would you trust MOST to help you decide whether you would get a COVID-19 vaccine, if one becomes available?	Your doctor or healthcare provider	Donald Trump; Anthony Fauci; Your state's governor; Local public health authority	Friends or family	Your pastor, priest, or other religious leader	.	Other; Joe Biden

Table S13 presents question wording and answer options used in Figure 3 to get the percentage of respondents mentioning each actor or institution that they would trust to decide whether to get the COVID-19 vaccine. Columns 3-8 show the answer options that were recoded in each category. Answer options are separated by a semicolon.
