# Supplementary material for: COVID-19 vaccine acceptance and hesitancy in low- and middle-income countries
Source: Nat Med. 2021 Jul 16;27(8):1385–94. doi: 10.1038/s41591-021-01454-y (PMC8363502; doi:10.1038/s41591-021-01454-y)
Supplement: Supplementary file 2 — Reporting Summary [file 41591_2021_1454_MOESM2_ESM.pdf]

nature research

Corresponding author(s): Saad B. Omer and Ahmed Mushfiq Mobarak

Last updated by author(s): Jul 12, 2021

Reporting Summary

Nature Research wishes to improve the reproducibility of the work that we publish. This form provides structure for consistency and transparency in reporting. For further information on Nature Research policies, see our Editorial Policies and the Editorial Policy Checklist.

Statistics

For all statistical analyses, confirm that the following items are present in the figure legend, table legend, main text, or Methods section.

n/a	Confirmed	
☐	☒	The exact sample size (*n*) for each experimental group/condition, given as a discrete number and unit of measurement
☐	☒	A statement on whether measurements were taken from distinct samples or whether the same sample was measured repeatedly
☐	☒	The statistical test(s) used AND whether they are one- or two-sided<br>*Only common tests should be described solely by name; describe more complex techniques in the Methods section.*
☐	☒	A description of all covariates tested
☒	☐	A description of any assumptions or corrections, such as tests of normality and adjustment for multiple comparisons
☐	☒	A full description of the statistical parameters including central tendency (e.g. means) or other basic estimates (e.g. regression coefficient) AND variation (e.g. standard deviation) or associated estimates of uncertainty (e.g. confidence intervals)
☐	☒	For null hypothesis testing, the test statistic (e.g. *F*, *t*, *r*) with confidence intervals, effect sizes, degrees of freedom and *P* value noted<br>*Give P values as exact values whenever suitable.*
☒	☐	For Bayesian analysis, information on the choice of priors and Markov chain Monte Carlo settings
☒	☐	For hierarchical and complex designs, identification of the appropriate level for tests and full reporting of outcomes
☒	☐	Estimates of effect sizes (e.g. Cohen's *d*, Pearson's *r*), indicating how they were calculated

Our web collection on statistics for biologists contains articles on many of the points above.

Software and code

Policy information about availability of computer code

Data collection	No software was used for data collection
Data analysis	Authors used R 4.0.4. All the relevant packages and ad hoc functions developed for the analysis can be found in the replication material https://github.com/wzb-ipi/covid_vaccines_nmed

For manuscripts utilizing custom algorithms or software that are central to the research but not yet described in published literature, software must be made available to editors and reviewers. We strongly encourage code deposition in a community repository (e.g. GitHub). See the Nature Research guidelines for submitting code & software for further information.

Data

Policy information about availability of data

All manuscripts must include a data availability statement. This statement should provide the following information, where applicable:

- Accession codes, unique identifiers, or web links for publicly available datasets
- A list of figures that have associated raw data
- A description of any restrictions on data availability

Individual participant data (de-identified) that underlie the results reported in this article, analytic code and replication files are available to no end date for anyone who wishes to access the data and use it for any purpose here https://github.com/wzb-ipi/covid_vaccines_nmed. A replication exercise is available here https://wzb-ipi.github.io/covid_vaccines_nmed/.

Table 2 in the main text uses data from the Wellcome Global Monitor 2018, available here https://beta.ukdataservice.ac.uk/datacatalogue/studies/study?id=8466#!/details and from the WHO-UNICEF immunization coverage estimates available here https://apps.who.int/immunization_monitoring/globalsummary/timeseries/tswucoveragedtp3.html

Subsection COVID-19 Case History for Countries Included in the Study in the Methods section quotes data from Johns Hopkins Coronavirus Reource Center available here https://coronavirus.jhu.edu/data

Field-specific reporting

Please select the one below that is the best fit for your research. If you are not sure, read the appropriate sections before making your selection.

☐ Life sciences ☒ Behavioural & social sciences ☐ Ecological, evolutionary & environmental sciences

For a reference copy of the document with all sections, see nature.com/documents/nr-reporting-summary-flat.pdf

Behavioural & social sciences study design

All studies must disclose on these points even when the disclosure is negative.

Study description

Quantitative analysis of standardized survey responses from 15 studies to estimate willigness to take a COVID-19 vaccine, the reasons for acceptance and hesitancy and trusted sources of information about COVID-19 vaccine in low and middle income countries

Research sample

15 studies in ten LMICs in Africa (Burkina Faso, Mozambique, Nigeria, Rwanda, Sierra Leone, Uganda), Asia (India, Nepal, Pakistan), and Latin America (Colombia), and upper-middle-income country (Russia) and the United States. Table 3 summarizes the time span, geographic scope, sampling methodologies and survey modalities of all 15 studies. A detailed description of each study is included below under "Sampling strategy".

Sampling strategy

The Burkina Faso, Colombia, Rwanda and Sierra Leone survey 1 samples were drawn from the RECOVR studies implemented by IPA. The target population for these studies comprised all adults with mobile phone numbers in the country, based on national communications authority number allocation plans. The sampling frame consisted of all mobile phone numbers in the countries. Numbers were called via RDD, stratified by mobile network operator market share for a two-round panel survey. In Burkina Faso, the sample included 977 respondents contacted in the second round of a panel of 1,383. In Colombia, the sample included 1,012 respondents contacted in the second round of a panel of 1,507. In Rwanda, the sample included 1,355 respondents contacted in the second round of a panel of 1,480. In Sierra Leone survey 1, the sample included 1,070 respondents contacted in the second round of a panel of 1,304. Post-stratification weights were computed to adjust for differential attrition between the first and second rounds of the RDD panel, weighting on gender, region and educational attainment.

In the 'India, Coping with COVID-19 in Slums' subnational sample was drawn from research undertaken by the Nova School of Business and Economics, The Institute for Fiscal Studies, and the University of St Andrews. The target population was a random subset of slum populations in Lucknow and Kanpur, Uttar Pradesh, India. Socio-economic variables were only collected for a representative sample of the population relying on community toilets or open defecation to fulfill their sanitation needs. The study design was a randomized controlled trial, with complete census of households within 142 slums (carried out from September to December 2017) and a series of household and caretaker surveys, objective measurements, incentivized behavioral measurements and a structured community activity, collected for a subset of 100 slums between April 2018 and September 2019. The catchment areas of community toilets were randomly allocated to two interventions. The first intervention aimed at community toilet improvements by offering caretakers the choice of a grant to be spent for improvements in the facility. Following the grant, caretakers were offered a large financial reward conditional on the cleanliness of the facility. The second intervention added to this community toilet improvement awareness creation through face-to-face information sessions, leaflets, monthly reminders using voice messages sent to mobile phones, and posters hung in the community toilets. A two-step sampling was applied: study households from the main study sample were first sampled, then households from the whole slum population were added. The baseline ran from June to July 2020, follow-up 1 ran from October to November 2020, and follow-up 2 ran from 16 December 2020 to 18 January 2021. The sample size was 3,991 households, with a mean of 28 households per cluster (142). Baseline non-response was 25%, and the attrition rate from baseline to follow-up (1 and 2) was 13%. The study included 1,277 randomly selected replacement households for follow-up (1 and 2). Sampling weights are included.

The Mozambique Subnational sample, implemented by the International Growth Center and the Nova School of Business and Economics targeted microentrepreneurs in urban markets of Maputo and household heads from the province of Cabo Delgado. The initial data was collected in-person in two different studies. For microentrepreneurs in Maputo, the data was collected between October 2013 and April 2014 (baseline), and between July and November 2015 (endline). For household heads in Cabo Delgado, the data was collected in-person between August and September 2016 (baseline), and between August and September 2017 (endline). The first study was dedicated to analyzing the impacts of interventions targeting microentrepreneurs in urban markets on financial inclusion and literacy. The second study focused on the role of information to counteract the political resource curse after a substantial natural gas discovery. The first initial sample was selected by in-field random sampling in 23 urban and periurban markets in Maputo and Matola. Stratification was based on the gender of the respondent and on the type of establishment (stall vs. store). The second initial sample was selected to be representative of 206 communities in the province of Cabo Delgado, randomly drawn from the list of all 421 polling locations in the sampling frame, stratified on urban, semiurban, and rural areas. The sample includes 554 microentrepreneurs from Maputo and 308 households from Cabo Delgado.

The Nepal Western Terai Panel Survey (WTPS) Subnational sample was implemented by researchers from Yale University and the Yale Research Initiative on Innovation and Scale (Y-RISE). Its target population was rural households in the districts of Kailali and Kanchanpur. Initial baseline data was collected in-person in July of 2019, and 5 rounds of phone survey data were collected between August 12, 2019 and January 4, 2020. The phone survey sample includes 2,636 rural households in the districts of Kailali and Kanchanpur, which represent the set of households that responded to phone surveys from an original sample of 2,935 households.

This sample was constructed by randomly sampling 33 wards from 15 of the 20 sub-districts in Kailali and Kanchanpur and selecting a random 97 villages from within those wards. At the time of baseline data collection in July of 2019, 7 of these 97 villages were dropped from the sample due to flooding. Households belong to the bottom half of the wealth distribution in these villages, as estimated by a participatory wealth ranking exercise with members of the village. The sample included in this study includes 1,392 households.

The Nigeria Subnational sample was implemented by researchers from WZB Berlin Social Science Center and the University of Illinois Chicago. The target population included Christian and Muslim men and women, age 18 and above, living in Kaduna state, Nigeria. Initial data was collected from a subset of the sample in December 2019 (in person survey) and July - Aug 2020 (phone survey) as part of an experiment testing the effects of a brief radio program on inter-religious animus. A random walk procedure and random sampling were used within households to recruit a representative sample of adults in Kaduna town. The rest of the sample was recruited for the study in Aug 2020 by purchasing phone lists for residents of Kaduna State. The subset of the sample in the radio study was randomly assigned to listen to a brief radio program on one of the following topics: (1) an inter-religious storyline, (2) an intra-religious storyline, and (3) a message about maintaining safe health practices. All respondents in the sample participated in a study examining the effect of viewing an inter-religious storyline unfolding over a full season of a popular TV drama, Dadin Kowa. The season aired from Aug - Oct 2020. A third of the sample were encouraged to watch Dadin Kowa, a third were encouraged to watch the TV station Africa Magic Hausa at the same time Dadin Kowa aired, and a third were in the treatment-as-usual group. All participants received a weekly incentivized SMS quiz from Aug Oct 2020. The survey from which this data is drawn is not primarily about COVID-19, but was designed as an endline survey to follow the TV drama intervention described above. The goal of the COVID-19 survey is to measure a range of attitudinal outcomes related to Christian-Muslim relations (including prejudice, intergroup threat perceptions, dehumanization, and support for the use of violence, among others). We included nine of the standardized COVID-19 vaccine-related questions collected specifically for this vaccine acceptance study in the final module of the endline survey. 950 respondents in the sample were recruited in person through a random sampling procedure in the Kaduna metropolitan area (pre-COVID). The remaining 1,700 respondents were recruited into the study over the phone from lists of phone numbers of Kaduna state residents that were purchased from a private vendor. All 1,834 individuals who completed the endline survey are included.

The Pakistan survey 1—Sheikhupura Police Study Sample—was implemented by the Institute of Development and Economic Alternatives, Lahore University of Management Science, London School of Economics, and Princeton University (Pakistan 1). The target population is a representative sample of adults from 108 of 151 police beats in Sheikhupura and Nankana districts of Punjab Province. The survey involved calls to all households in the stratified random sample for the policing study midline survey. The sampling frame included households in Sheikhupura and Nankana districts, and the sample includes 1,473 respondents. Post-stratification weights are computed to adjust for the sampling process, which involved stratifying first on 27 police stations, then within each police station on beats, then probability proportional to size (PPS) within beats using Asiapop population data.

The Pakistan Economic Vulnerability Assessment (EVA) subnational sample (Pakistan survey 2), was implemented by the Lahore University of Management Studies and targeted all possible mobile phone numbers in the province of Punjab generated based on the local mobile phone number structure in Pakistan. The survey involved making calls to individuals in Punjab based on random digit dialing. The sample includes 1,492 respondents.

The Russian Federation, Research on COVID-19 in Russia's Regions (RoCiRR) Subnational sample, was implemented by the International Center for the Study of Institutions and Development (HSE University, Moscow, Russia) and Economics Department of Ghent University and Columbia University. The target population was adult internet users who reside in one of 61 federal subjects (federal cities, oblasts, republics, krais and autonomous okrug) of Russia. The regions included in the study are the following Republics (Bashkortostan, Karelia, Komi, Mariy El, Mordovia, Tatarstan, Udmurtia, Chuvashia), Krais (Altai, Krasnodarsky, Krasnoyarsky, Permsky, Primorsky, Stavropolsky, Khabarovsky), Oblasts (Arkhangelsk, Astrakhan, Belgorod, Bryansk, Vladimir, Volgograd, Vologda, Voronezh, Ivanovo, Irkutsk, Kaliningrad, Kaluga, Kemerovo, Kirov, Kostroma, Kurgan, Kursk, Leningrad, Lipetsk, Moscow, Murmansk, Nizhny Novgorod, Novgorod, Novosibirsk, Omsk, Orenburg, Orel, Pskov, Penza, Rostov, Ryazan, Samara, Saratov, Sverdlovsk, Smolensk, Tambov, Tver, Tomsk, Tula, Tyumen, Ulyanovsk, Chelyabinsk, Yaroslavl), as well as Moscow, Saint Petersburg, and Khanty-Mansiysk Autonomous Okrug – Ugra. The remaining 24 federal subjects were excluded from the study due to inability to enroll sufficient sample size with desired characteristics (sample size, age, gender and education group composition) and because they account for less than 14% of the total adult population of Russia. The study was designed to measure the impact of pandemics on Russians, mostly those who live in cities with more than 100,000 residents. It contains a number of questions on the personal experience, norms and values, trust in government institutions, provision of social services, and mass media use. Region and geolocality of every respondent are recorded. In total 25,558 respondents received the module on vaccine acceptance. The sample was enrolled from the pool of Russian online survey company OMI (Online Market Intelligence). The sampling was specifically targeted at having a minimum of 150 respondents in each of the 61 regions and including respondents from all the main age and gender groups within each region. Respondents were also selected so that at least 40% of respondents did not have higher education, in accordance with higher education rates in Russia. Out of 25,558 recruited respondents, 22,125 completed the survey. Among 22,125 respondents who completed the survey, 20,821 were enrolled from the general pull of the survey company respondents, while the remaining 1,304 respondents were enrolled among residents of cities with populations below 100,000 and rural areas. Post-stratification weights are computed to match marginal distributions of age, gender and education among the adult population of Russia with target proportions coming from the 2019 Yearbook and 2015 Microcensus released by Russian Federal Bureau of National Statistics (Rosstat).

The Sierra Leone Rural Electrification (SLRE) project sample (Sierra Leone survey 2) included towns that are candidates for rural electrification. This nation-wide sample was implemented by the International Growth Centre (IGC), Wageningen University & Research, Yale Research Initiative on Innovation and Scale (Y-RISE), WZB Berlin Social Science Center and Columbia University. The study included households in 195 rural towns across all 14 districts of Sierra Leone. Of these, 97 villages were selected to benefit from an electrification program. For the original study, initial baseline data was collected during late 2019 and early 2020 as part of a study to assess the impact of Rural Electrification in rural towns in Sierra Leone. The Government of Sierra Leone (GoSL) in collaboration with the United Nations Office for Project Services (UNOPS) and international donors is implementing the Rural Renewable Energy Project (RREP). In its first wave, during 2017, the project provided stand-alone solar photovoltaic powered mini-grids to 54 communities across the country. Construction of mini-grids in 43 further towns is ongoing. In RREP communities, engineers construct 6kW–36kW power mini-grids that provide reliable power year-round. Electricity is free for schools and clinics. Residential and commercial users can acquire connections from commercial operators. Household data was collected in 195 towns across all 12 districts of Sierra Leone. The GoSL selected 97 towns with (planned) mini-grids. We used Propensity Score Matching to

select 98 control communities. Within communities, respondents were randomly selected from a census roster stratified by occupation status of farmers, business owners and other occupations [47 percent, 47 percent and 7 percent]. In each village, the intended sample was 43 households (20 farmers, 20 businesses, 3 others). Data was collected during June–July (108 communities) and November–December 2019 (87 communities). If a household on the sampling list was not available on the village visit day, we had a randomly sampled list of replacement households to survey. The replacement household would be the same occupation as the sampled household would have been so the sample ratio of 20-20-3 still held in each community. The goal of the COVID-19 survey was to assess households' degree of economic vulnerability in the face of the COVID-19 pandemic. The COVID-19 survey data comprises 2,110 respondents from 186 towns from the original baseline survey. Phone surveys were attempted to all 195 rural communities from the baseline survey. The total baseline household sample comprised 7047 respondents. We recontacted all baseline respondents that listed a phone number (4,594 respondents) and obtained informed consent for the phone survey. We implemented several waves of the phone survey, recontacting a respondent about every month. In wave 7, we added questions related to Vaccine Acceptability. Data collection took place between October 7 2020 and January 20, 2021with 2,110 respondents, in 186 towns for a tracking rate of 46 percent.

The Uganda survey 1 subnational sample, was implemented by the International Growth Center, Trinity College Dublin, Stockholm School of Economics and Misum, Institute for International Economic Studies, Stockholm University. The target population was women from semi-rural and rural villages across 13 districts in Uganda (Iganga, Kayunga, Mbale, Mityana, Apac, Dokolo, Gulu, Adjumani, Koboko, Maracha, Nebbi, Soroti, Kumi). For the original study, initial baseline data was collected in 2016 as part of a large cluster randomized controlled trial, with the aim of selecting households likely to have children during the study period. Four criteria for selection were thus used, in descending order of importance: the household has a woman that is currently pregnant, or aged 16-30 years old, with a young child less than three years old, and/or married (formally or informally). In each household, the respondent was chosen as the female household head or the primary female health care giver of the household if the household head could not be found. The COVID-19 survey data was collected through multiple rounds of phone surveys. The variable measuring age was constructed by approximation, using the baseline data from 2016 and adding 4 years to the 2016 measure. When the baseline respondent was replaced, the initial age information was deleted. Households were selected within 500 clusters (the village of the household). Out of 2,743 respondents, 1752 were included, provided that they answered the main question about vaccine uptake.

The Uganda survey 2 subnational sample, was implemented by WZB Berlin Social Science Center and Columbia University, NYU Abu Dhabi, and Innovations for Poverty Action (IPA). The target population was all residents of Kampala who are Ugandan citizens, above the age of 18, and agree in principle to attend a short citizen consultative meeting. For the original study, baseline data was collected between July and October 2019 for an intervention that randomized citizen attendance to a set of 188 consultative meetings organized across Kampala. The meetings were organized to collect citizen preferences for the design of a forthcoming municipal citizen charter. The study also aimed to assess patterns of political inequality in meeting participation, dynamics, and outcomes, as well as study the subsequent effects on prosociality of being incorporated in this participatory process. 1/3 of the sample was randomly allocated to control, while 2/3 of respondents were invited to attend a consultative meeting. The consultations took place between November 2019 and February 2020 across Kampala divisions. The intervention consisted of attendance at the consultative meeting organized a few months after baseline data collection. A further randomization allocated ½ of the invited participants to a meeting moderated by a local bureaucrat, while the remaining ones attended a meeting moderated by a neutral discussion leader. The COVID-19 survey sample comprises the 2,189 respondents to the baseline who were selected on the basis of their residence in the city. Having received permission to re-contact these individuals, we coordinated a 3-wave panel throughout the summer and fall of 2020, with respondents contacted via phone. The goal was to assess households' degree of economic vulnerability in the face of the COVID-19 pandemic and respondents' evaluations of performance of political actors in tackling the pandemic. The 2,189 respondents to the baseline were randomly selected from a sampling frame of all buildings in Kampala, for which information about their geographical coordinates was available. After randomly selecting a set of candidate structures, interviewers sampled respondents from the subset of structures that were residential. Of the 2,189 respondents which we aimed to contact, we were able to reach 1,333 in Wave 1, 1,289 in Wave 2, and 1,366 in Wave 3. Wave 3 contained the COVID-19 vaccine module presented in this analysis.

The United States of America nation-wide sample was implemented by WZB Berlin Social Science Center, Cornell University, and Tufts University. The target population was a nation-wide sample of adult internet users recruited through the market research firm Lucid. This survey was part of a panel study on attitudes toward COVID-19 technologies and public health surveillance. The Lucid Marketplace is an automated marketplace that connects researchers with willing online research participants. Lucid partners with a network of companies that maintain relationships with research participants by engaging them with research opportunities. While Lucid does not provide probability samples of the U.S. adult population, its quota samples approximate the marginal distributions of key demographic characteristics. Recent validation exercises have found that Lucid samples approximate nationally representative samples in terms of demographic characteristics and survey experiment effects. The sample includes 1,959 individual online surveys. In the main question regarding intention to take the vaccine, approximately 10% of respondents (184) did not answer. Post-stratification weights are computed to match marginal population distributions of income, age, education, gender, race and region among the US adult population, with target proportions based on the 2018 American Community Survey.

Data collection

All surveys of the LMIC samples were conducted via telephone to minimize in-person contact and comply with local government social distancing guidelines. Interviews were conducted by local staff in each country in local language(s). Surveying by phone made rapid, large-scale data collection possible. Surveys lasted approximately 15 to 40 minutes. Only enumerators and subjects were present during the survey.

Surveys from Russia and the United States were conducted online, as detailed above.

Across studies, we asked respondents, "If a COVID-19 vaccine becomes available in [your country], would you take it?". If the respondent answered yes to this question, we followed up with the question, "Why would you take it? [the COVID-19 vaccine]". If the respondent said they would not be willing to take the vaccine, we followed up with the question, "Why would you not take it? [the COVID-19 vaccine]". Finally, regardless of their expressed willingness to take the vaccine, we asked about actors and institutions that would be most influential in their decision: "Which of the following people would you trust MOST to help you decide whether you would get a COVID-19 vaccine, if one becomes available?" following. To examine heterogeneity across demographic strata, we collected information about gender, age, and education. Slight variations in question wording and answer options across studies are

	documented in Supplementary Tables 9-12 .
Timing	From Survey data were collected between June 2020 and January 2021. The time span of each individual study is summarized in Table 3.
Data exclusions	No data was excluded from the analyses
Non-participation	All participants consented to participation in the research verbally or via online forms (USA and Russia only). Subjects were free to withdraw consent or leave the survey at any point. We include in the analyses all subjects that answer at least the first question ("If a COVID-19 vaccine becomes available in [your country], would you take it?").
Randomization	Experimental manipulation is not relevant in this study. Nonetheless, we conducted subgroup analyses by gender, age and education level and reported differences between groups. Covariates (age, gender, education) were collected by individual teams. In the study we recoded them into common categories in order to maximize harmonization. For age, we selected cut-offs below 25, between age 25 and 54, and 55 years old and above, closely following the age breakdown proposed by recent work on COVID-19 vaccine acceptance and considering the age distribution of the populations of the countries in our samples, which limited data collection for older age cohorts. For education, we divided the sample between respondents who had completed secondary school and those that had not. Gender was coded as a binary variable, and subjects divided between male and female.

Reporting for specific materials, systems and methods

We require information from authors about some types of materials, experimental systems and methods used in many studies. Here, indicate whether each material, system or method listed is relevant to your study. If you are not sure if a list item applies to your research, read the appropriate section before selecting a response.

Materials & experimental systems

n/a	Involved in the study
☒	☐ Antibodies
☒	☐ Eukaryotic cell lines
☒	☐ Palaeontology and archaeology
☒	☐ Animals and other organisms
☐	☒ Human research participants
☒	☐ Clinical data
☒	☐ Dual use research of concern

Methods

n/a	Involved in the study
☒	☐ ChIP-seq
☒	☐ Flow cytometry
☒	☐ MRI-based neuroimaging

Human research participants

Policy information about studies involving human research participants

Population characteristics	See above and Methods section
Recruitment	All studies analyzed in this manuscript were conducted via phone or online (USA, Russia) between 17 June, 2020 and January 18, 2020. Details on recruitment of individual studies can be seen above and in the Methods section.
Ethics oversight	Each of the individual studies obtained IRB approval independently. The Burkina Faso study was approved via IPA IRB Protocol 15608, and the Burkina Faso Institutional Ethics Committee for Health Sciences Research, approval A13-2020. The Colombia study was approved via IPA IRB Protocol 15582. The India study was approved via the London School of Economics (REC ref. 1132). The Mozambique study was approved by Universidade Nova de Lisboa. The Nepal study was approved via Yale University IRB Protocol 2000025621. The Nigeria study was approved via the IRB at the University of Pennsylvania (Protocol 834548). The Pakistan Survey 1 was approved via Princeton University IRB Protocol 7250. Pakistan Survey 2 was approved via Lahore University of Management Sciences IRB Protocol LUMS-IRB/07012020SA. The Rwanda RECOVR study was approved via IPA IRB Protocol 15591, Rwanda National Institute for Scientific Research permit No.0856/2020/10/NISR; and Rwanda National Ethics Committee approval No.16/RNEC/2020. The Russia study was approved via Columbia University IRB Protocol IRB-AAAT4453. Sierra Leone Study 1 was approved via IPA IRB Protocol 15592 and Sierra Leone Ethics and Scientific Review Committee (no number provided, letter available upon request), and Sierra Leone Study 2 was approved via Sierra Leone Ethics and Scientific Review Committee (SLERC 2904202) and Wageningen University (24062020). Uganda Study 1 was approved via Mildmay Uganda Research Ethics Committee (protocol number 0109-2015). Uganda Study 2 was approved via IPA IRB (protocol number 15018), WZB Berlin Social Science Center Ethics Review Board (protocol number 2020/0/91), NYU Abu Dhabi IRB (protocol number HRPP-2020-64), MIT Committee on the Use of Humans as Experimental Subjects (protocol number 2005000155), and by the Mildmay Uganda Research Ethics Committee (protocol number 0604-2019). The USA study was approved via Cornell University IRB under Protocol number 2004009569.

Note that full information on the approval of the study protocol must also be provided in the manuscript.
